# Elastodynamic image forces on dislocations

**DOI:** 10.1098/rspa.2015.0433

**Published:** 2015-09-08

**Authors:** Beñat Gurrutxaga-Lerma, Daniel S. Balint, Daniele Dini, Adrian P. Sutton

**Affiliations:** 1 Department of Mechanical Engineering, Imperial College London, London SW7 2AZ, UK; 2 Department of Physics, Imperial College London, London SW7 2AZ, UK

**Keywords:** dislocation, edge dislocation, screw dislocation, image force, elastodynamic

## Abstract

The elastodynamic image forces on edge and screw dislocations in the presence of a planar-free surface are derived. The explicit form of the elastodynamic fields of an injected, quiescent screw dislocation are also derived. The resulting image forces are affected by retardation effects: the dislocations experience no image force for a period of time defined by the arrival and reflection at the free surface of the dislocation fields. For the case of injected, stationary dislocations, it is shown that the elastodynamic image force tends asymptotically to the elastotatic prediction. For the case of injected, moving dislocations, it is shown that the elastodynamic image force on both the edge and the screw dislocations is magnified by inertial effects, and becomes increasingly divergent with time; this additional effect, missing in the elastostatic description, is shown to be substantial even for slow moving dislocations. Finally, it is shown that the elastodynamic image force of an edge dislocation moving towards the surface at the Rayleigh wave speed becomes repulsive, rather than attractive; this is suggestive of instabilities at the core of the dislocation, and likely resonances with the free surface.

## 1. Introduction

In the presence of a free surface, dislocations experience a force that drives them towards it; this results in the minimum energy configuration for the material, whereby dislocations become steps on its free surface [1]. Linear elasticity provides an accurate description of the magnitude of the attractive force between a dislocation and a free surface, which is usually called the *image force* because of the ‘*image dislocation*’ construction employed in its derivation. This construction, analogous to the image charge construction employed in electromagnetism, computes the force as if it were due to an image dislocation, equidistant from the surface but outside the material, and of the same magnitude as the original dislocation, but of opposite sign.

In the past, image forces have been calculated for a wide range of different geometrical configurations [1]. However, to the authors' knowledge, all previous attempts to derive image forces on dislocations have been elastostatic, i.e. have considered only static equilibrium conditions for both the dislocation and its medium.

The elastostatic derivations identify the presence of an attractive force that usually grows in inverse proportion to the distance between the dislocation and the free surface, *l* [1]. For instance, for straight dislocations parallel to a planar surface in an elastic half space, it is found that

1.1 $$F_{x}=\frac{\mu B^{2}}{4\pi }\left[\frac{1}{l}\right]\;\text{for}\;\text{screw}\;\text{and}\;F_{x}=\frac{\mu B^{2}}{4\pi (1-\nu )}\left[\frac{1}{l}\right]\;\text{for edge},$$

where *B* is the magnitude of the Burgers vector, *μ* the shear modulus and *ν* Poisson's ratio, and *F*_*x*_ is the image force in the glissile direction per unit length.

This article derives the elastodynamic image forces experienced by both straight screw and edge dislocations, moving and quiescent, in the presence of a planar-free surface. Time is included as an explicit field variable in the linear elastic description of the problem and, as a result, inertial effects affecting both the dislocation and the medium are accounted for.

**Figure 1.**
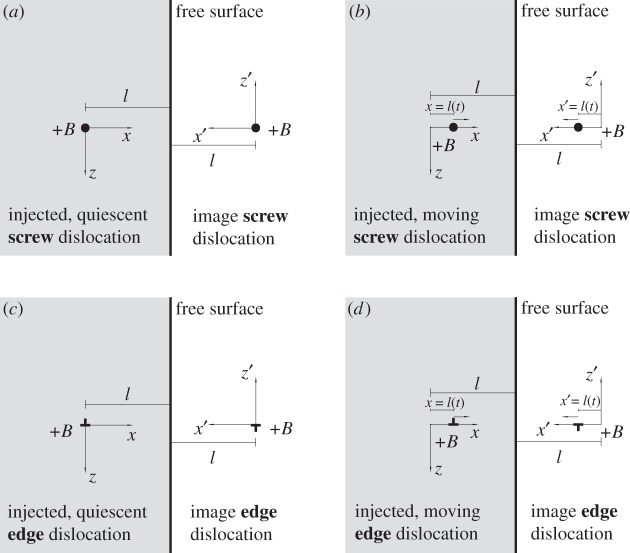
(*a*–*d*) Geometrical configurations of edge and screw dislocations for the derivation of the image forces.

The configurations studied in this article are shown in figure 1. Section 2 considers the case of an injected, quiescent (non-moving) screw dislocation; §3 that of an injected, moving screw dislocation; §4 the general formulation used for tackling the case of edge dislocations; §5 that of an injected, quiescent (non-moving) edge dislocation; §6 that of an injected, moving edge dislocation.

Throughout the article, and unless otherwise stated, the Cartesian coordinate systems shown in figure 1 will be adopted: the dislocation's injection site is located at the origin of the (*x*,*z*) basis; the image dislocation's injection site is located at (*x*=2*l*,*z*=0) with respect to that coordinate system. The image dislocation can be simulated as a negative dislocation with respect to the (*x*,*z*) basis or, equivalently, as a positive dislocation with respect to a rotated Cartesian coordinate system (*x*′,*z*′), with its origin at the image dislocation's injection site, whereby *x*′↦*x*−2*l* and *z*′↦−*z*. This is advantageous because in that way the moving image dislocation advances along the positive direction of its local *x*′ axis. All Cartesian bases are considered to be right-handed. Accordingly, a positive dislocation has its Burgers vector oriented along the positive direction of the out-of-plane *y*-axis for screw dislocations, and the *x*-axis for edge dislocations.

## 2. Image force on an injected, quiescent screw dislocation

Consider the straight, infinite screw dislocation shown in figure 1*a*. The dislocation is injected (created) at time *t*=0 at a distance *l* from a planar-free surface in an elastic half space. The dislocation line, and the Burgers vector, are oriented along the *y*-axis in an elastic half space; the dislocation line is perpendicular to the surface's normal vector. The free surface imposes the boundary condition that

$$\sigma_{ij}n_{j}=0,$$

where *n*_*j*_ is the surface's normal vector. As will be seen below, the elastodynamic fields of a screw dislocation consist solely of its shear components, which are therefore required to vanish at the surface.

As in the elastostatic analogue, the boundary conditions can be satisfied by considering an *image screw dislocation*, of the same magnitude as the original but of opposite sign, which is shown in figure 1*a*; the image dislocation is also injected at time *t*=0 at a distance *l* away from the free surface. The elastodynamic fields of the image dislocation can be derived in the same way as for the injected dislocation's.

### (a) Elastodynamic fields of an injected, quiescent screw dislocation

The injected, quiescent screw dislocation was first studied by Jokl *et al.* [2], although they did not provide an explicit expression for its elastodynamic fields; these fields are therefore derived here.

Following the coordinate system shown in figure 1*a*, consider the injected dislocation at position *x*=0; the free surface is at position *x*=*l*, and the image dislocation is at position *x*=2*l*.

Consider the governing equation [2]:

2.1 $$\frac{\partial^{2}u_{y}}{\partial x^{2}}+\frac{\partial^{2}u_{y}}{\partial z^{2}}=b^{2}\frac{\partial^{2}u_{y}}{\partial t^{2}},$$

where *b*=1/*c*_*t*_ is the transverse slowness of sound, and subject it to the following boundary condition:

2.2 $$u_{y}=BH(x)H(t)$$

and

2.3 $$\sigma_{zz}=0,$$

where *B* is the magnitude of the Burgers vector, *H*(⋅) the Heaviside function and *u*_*y*_ the displacement component in the *y*th direction.

Define the following Laplace transforms, applied successively to any function *f*(*x*,*z*,*t*):

2.4 $$\hat{f}(x,z,s)=\int_{0}^{\infty }f(x,z,t)\;e^{-st}\;\text{d}t$$

and

2.5 $$F(\lambda ,z,s)=\int_{0}^{\infty }\hat{f}(x,z,s)\;e^{-s\lambda x}\;\text{d}x.$$

Applying these two transforms to equation (2.1), one obtains

2.6 $$\frac{\partial^{2}U_{y}}{\partial z^{2}}=(b^{2}-\lambda^{2})s^{2}U_{y},$$

the solution of which will be of the form

2.7 $$U_{y}(\lambda ,z,s)=C\cdot e^{-s\beta z},$$

where *β*^2^=*b*^2^−λ^2^ and *C* is an integration constant that can be found from the boundary conditions as follows.

Applying the successive Laplace transforms to the displacement boundary condition (equation (2.2)), one obtains

2.8 $$U_{y}(\lambda ,0,s)=\frac{B}{2\lambda s^{2}},$$

whereby the integration constant must be *C*=*B*/2λ*s*^2^. Hence, the general solution is of the form

2.9 $$U_{y}(\lambda ,z,s)=\frac{B}{2\lambda s^{2}}\;e^{-s\beta z}.$$

This solution can be inverted employing the Cagniard–de Hoop technique (see [2–5]).

Only the *σ*_*xy*_ stress component is of interest; it is given *σ*_*xy*_=*μu*_*y*,*x*_. Following that, the transformed stress field component is given by

2.10 $$\Sigma_{xy}=\frac{\mu B}{2s}\;e^{-s\beta z},$$

where $\Sigma_{xy}=L_{x}\{{\hat{\sigma }}_{xy}\}=L_{x}\{L_{t}\{\sigma_{xy}\}\}$.

The first inverse Laplace transform is the following integral:

2.11 $${\hat{\sigma }}_{xy}=\frac{1}{2\pi i}\int_{-i\infty }^{i\infty }\frac{\mu B}{2s}\;e^{-s\beta z}\;e^{\lambda sx}s\;\text{d}\lambda .$$

By appropriate change of variable, the Bromwich integral can be given the form of a forward Laplace transform. Thus, call *τ*=*βz*−λ*x*. Invoking Cauchy's theorem and Jordan's lemma, the value of the integral along the (−i∞,i∞) integration contour can be equated to that along the hyperbola branches defined by $\lambda_{+}=(-\tau x+iz\sqrt{\tau^{2}-(x^{2}+z^{2})b^{2}})/(x^{2}+z^{2})$. The hyperbola branch corresponds with an integration path from $\tau =b\sqrt{x^{2}+z^{2}}$ to τ→∞ with respect to *τ*.

This entails that the inverse Laplace transform can be rewritten as

2.12 $${\hat{\sigma }}_{xy}=\frac{\mu B}{2\pi }\int_{0}^{\infty }\text{Im}\left[\frac{\partial \lambda_{+}}{\partial \tau }\right]H(\tau -b\sqrt{x^{2}+z^{2}})\;e^{-s\tau }\;\text{d}\tau ,$$

which is the Cagniard form of the solution. Upon applying the inverse Laplace transform in time,

2.13 $$\sigma_{xy}=\frac{1}{2\pi i}\int_{Br}\left\{\int_{0}^{\infty }\frac{\mu B}{2\pi }\;\text{Im}\left[\frac{\partial \lambda_{+}}{\partial \tau }\right]H(\tau -b\sqrt{x^{2}+z^{2}})\;e^{-s\tau }\;\text{d}\tau \right\}e^{st}\;\text{d}t,$$

it becomes clear that the solution is obtained by inspection as

2.14 $$\sigma_{xy}(x,z,t)=\frac{\mu B}{2\pi }\;\text{Im}\left[\frac{\partial \lambda_{+}}{\partial \tau }\right]H(\tau -b\sqrt{x^{2}+z^{2}}).$$

Expanding the imaginary part, the stress field is found to be

2.15 $$\sigma_{xy}(x,z,t)=\frac{\mu B}{2\pi }\frac{tx}{r^{2}\sqrt{t^{2}-b^{2}r^{2}}}H(t-br),$$

where *r*^2^=*x*^2^+*z*^2^.

It is interesting to note that in the t→∞ limit, this field converges to the elastostatic field of a screw dislocation

2.16 $$\sigma_{xy}(x,z)=\frac{\mu B}{2\pi }\lim_{t\to \infty }\frac{tx}{(x^{2}+z^{2})\sqrt{t^{2}-b^{2}(x^{2}+z^{2})}}H(t-b\sqrt{x^{2}+z^{2}})=\frac{\mu B}{2\pi }\frac{x}{x^{2}+z^{2}},$$

which is the expression of the static *σ*_*xy*_ stress field component of a screw dislocation [1].

Having found the field component, the image stress field exerted by the image dislocation will be (with respect to the coordinate system centred at the original dislocation),

2.17 $$\sigma_{xy}^{\mathrm{im}}(x,z,t)=\frac{\mu B}{2\pi }\frac{t(x-2l)}{({\left(x-2l\right)}^{2}+z^{2})\sqrt{t^{2}-b^{2}({\left(x-2l\right)}^{2}+z^{2})}}H(t-b\sqrt{{\left(x-2l\right)}^{2}+z^{2}}).$$

It is immediate to see that due to the symmetry of *σ*_*xy*_, at the surface (*x*=*l*) the image field cancels that of the dislocation

$$\begin{array}{rl} & \sigma_{xy}^{\mathrm{total}}(x=l,z,t)=\sigma_{xy}^{\mathrm{disloc}}(x=l,z,t)+\sigma_{xy}^{\mathrm{im}}(x=l,z,t)\\ & \;=\frac{\mu B}{2\pi }\left[\frac{tl}{(l^{2}+z^{2})\sqrt{t^{2}-b^{2}(l^{2}+z^{2})}}H(t-br)-\frac{tl}{(l^{2}+z^{2})\sqrt{t^{2}-b^{2}(l^{2}+z^{2})}}H(t-br)\right]=0, \end{array}$$

thereby satisfying the boundary condition that $\sigma_{xy}^{\mathrm{total}}=0$ at the free surface.

### (b) Image force

The image force exerted by the image dislocation on the real dislocation's line depends on the *σ*_*xz*_ component of stress, which can be derived using the same procedure described above.^1^ It is found to be

2.19 $$\sigma_{yz}(x,z,t)=\frac{\mu B}{2\pi }\frac{t[2t^{2}x-b^{2}(2x^{3}+x^{2}z+2xz^{2}+z^{3})]}{r^{2}\sqrt{t^{2}-b^{2}r^{2}}(2t^{2}-b^{2}r^{2})}H(t-br).$$

As before, this stress component converges to its elastostatic counterpart in the t→∞ limit.

The image component will then be, with respect to the (*x*,*z*)-axes,

2.20 $$\begin{array}{rl} \sigma_{yz}^{\mathrm{im}}(x,z,t) & =-\frac{\mu B}{2\pi }\frac{t[2t^{2}(x-2l)-b^{2}(2{\left(x-2l\right)}^{3}+{\left(x-2l\right)}^{2}z+2(x-2l)z^{2}+z^{3})]}{\left({\left(x-2l\right)}^{2}+z^{2})\sqrt{t^{2}-b^{2}({\left(x-2l\right)}^{2}+z^{2})}(2t^{2}-b^{2}({\left(x-2l\right)}^{2}+z^{2})\right)}\\ & \;\times H(t-b\sqrt{{\left(x-2l\right)}^{2}+z^{2}}). \end{array}$$

Thus, the image force on the injected, quiescent screw dislocation, given by *F*_*x*_(*t*)=*Bσ*_*yz*_(0,0,*t*), will be

2.21 $$F_{x}(t)=\frac{\mu B^{2}}{2\pi }\frac{t\sqrt{t^{2}-4b^{2}l^{2}}}{2lt^{2}-4b^{2}l^{3}}H(t-2bl).$$

As with the stress fields, in the t→∞ limit, the image force converges to its elastostatic counterpart. It is perhaps more revealing to rewrite the image force in terms of non-dimensional time *τ*=*t*/*bl*:

2.22 $$F_{x}(\tau )=\frac{\mu B^{2}}{2\pi }\left[\frac{1}{l}\right]\underset{\mathrm{Dynamic}\;\mathrm{contribution}}{\underbrace{\left[\frac{1}{2}\frac{\tau \sqrt{\tau^{2}-4}}{\tau^{2}-2}H(\tau -2)\right]}}.$$

One can easily identify two factors. The factor depending on *τ* in the second bracket is the elastodynamic contribution to the image force. For *t*>2*bl*, this elastodynamic contribution increases monotonically, and as t→∞, it tends to $\frac{1}{2}$. Hence, the image force asymptotically converges to its elastostatic counterpart, which as can be noted in equation (2.21) is proportional to 1/*l*. The term in the first bracket, 1/*l*, bears the same proportionality also found in the elastostatic image force.

**Figure 2.**
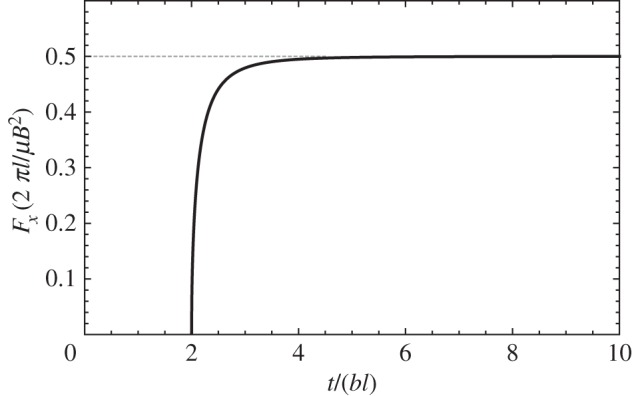
Evolution of the magnitude of the image force with time.

The evolution in the magnitude of the image force is shown in figure 2. As can be readily deduced from the elastodynamic contribution in equation (2.21), *F*_*x*_ takes no values prior to *t*=2*bl*. This is a result of the retardation principle underlying the elastodynamic formulation presented here. As a result of the finite propagation time of the elastodynamic shear waves, the elastic field of a dislocation injected at time *t*=0 at a distance *l* from the free surface will reach the free surface only at time *t*=*bl*; any reflected elastic wave incoming from the surface will require an additional *bl* to reach the dislocation, so until *t*=2*bl* the dislocation does not feel the presence of the free surface, and does not experience an image force.

## 3. Image force on an injected, moving screw dislocation

The solution for the image force of an injected, quiescent screw dislocation displays a behaviour very similar to that predicted by elastostatics. This should come as no surprise because in either case the fields describe a dislocation that does not move from its position. However, image forces are introduced to show that in the presence of a free surface, the dislocation will be attracted towards it as a way of minimizing the elastic energy of the system. Hence, one should expect the dislocation to move towards the surface.

The problem of the image fields of an injected, screw dislocation moving along the *x*-axis (figure 1*b*) is explored in this section.

### (a) Elastodynamic fields of a moving screw dislocation

The elastodynamic fields of a pre-existing, moving screw dislocation were derived by Markenscoff [4] by employing the Cagniard–de Hoop methodology. As argued by Gurrutxaga-Lerma *et al.* [3] when exploring the case of an injected, non-uniformly moving edge dislocation, the problem of the injected non-uniformly moving screw dislocation can be solved by superposition of two terms, arising from two separate boundary value problems.

(i) The mobile contribution, which in the screw case is of the form (see [4])
  3.1 $$u_{y}(x,0,t)=B[H(x-l(t))-H(x)]H(t).$$
(ii) The injection contribution
  3.2 $$u_{y}(x,0,t)=BH(x)H(t),$$
  which has in fact been solved in §2.

Here, *l*(*t*) is the *past history function*, a function that relates the position of the dislocation line to the instant in time *t* when it was occupied by the dislocation line. The governing equation is still equation (2.1). It is mathematically advantageous to transform the *H*(*x*−*l*(*t*)) function to take its inverse argument, *H*(*t*−*η*(*x*)), where *η*(*x*)=*l*^−1^(*t*) is the inverse of the past history function, i.e. the function that returns the instant in time when the dislocation line was at position *x*.

The solution procedure is analogous to that employed in the case of the injected, quiescent screw dislocation. It results in the following stress field components:

3.3 $$\sigma_{yz}^{\mathrm{mob}}(x,z,t)=\frac{\mu B}{2\pi }\frac{\partial }{\partial t}\int_{0}^{\infty }H(\tilde{t}-\tilde{r}b)\frac{t^{2}({\tilde{x}}^{2}-z^{2})-b^{2}{\tilde{r}}^{2}{\tilde{x}}^{2}}{{\tilde{r}}^{4}T_{b}}\;\text{d}\xi$$

and

3.4 $$\sigma_{xy}^{\mathrm{mob}}(x,z,t)=\frac{\mu B}{2\pi }\frac{\partial }{\partial t}\int_{0}^{\infty }H(\tilde{t}-\tilde{r}b)\frac{\tilde{x}z(b^{2}{\tilde{r}}^{2}-2{\tilde{t}}^{2})}{{\tilde{r}}^{4}T_{b}}\;\text{d}\xi ,$$

where $\tilde{x}=x-\xi$, $\tilde{t}=t-\eta (\xi )$, $\tilde{r}=\sqrt{{\tilde{x}}^{2}+z^{2}}$ and $T_{b}=\sqrt{{\tilde{t}}^{2}-{\tilde{r}}^{2}b^{2}}$. The order of integration and differentiation cannot generally be interchanged because some of the integration terms contain a square-root singularity along the integration path.

The special case when the dislocation's speed is constant, i.e. when *η*(*ξ*)=*d*⋅*ξ*, where *d*=1/*v* is the slowness of the dislocation and *v* its speed, allows for a direct integration of the solutions above, yielding:

3.5 $$\sigma_{yz}^{\mathrm{mob}}(x,z,t)=\frac{\mu B}{2\pi }\frac{b^{2}dr^{2}x^{2}-b^{2}tx(x^{2}+2z^{2})+dt^{2}(z^{2}-x^{2})+t^{3}x}{r^{2}\sqrt{t^{2}-b^{2}r^{2}}(-b^{2}z^{2}+d^{2}r^{2}-2dtx+t^{2})}H(t-rb)$$

and

3.6 $$\sigma_{xy}^{\mathrm{mob}}(x,z,t)=\frac{\mu B}{2\pi }\frac{z(b^{2}dr^{2}x-b^{2}tz^{2}-2dt^{2}x+t^{3})}{r^{2}\sqrt{t^{2}-b^{2}r^{2}}(-b^{2}z^{2}+d^{2}r^{2}-2dtx+t^{2})}H(t-rb).$$

As mentioned above, these fields must be superimposed with the fields of an injected screw dislocation derived in the previous section.

### (b) Image forces

Once the fields of a moving screw dislocation have been derived, the image forces can be computed for the case of a flat-free surface. As indicated in figure 1, the screw dislocation is moving towards a planar-free surface with a speed defined by the past history function *l*(*t*) (or its inverse, *η*(*x*)). As shown in figure 1*b*, and in direct analogy with the case of the quiescent dislocation, the image force can be computed by employing an image dislocation of opposite sign located outside the material, moving towards the surface with a past history function *l*(*t*) that prescribes the mirror-image of the motion of the actual dislocation.

As shown in figure 1*b*, the image dislocation is defined as a positive dislocation moving its local *x*′-axis.^2^ It must then be born in mind that the following coordinate transformations apply: *x*′↦*x*−2*l* and *z*′↦−*z*.

The free surface boundary condition requires that *σ*_*xy*_(*x*=*l*,*z*,*t*)=0. The system is made up of a dislocation and its image, the fields of which consist of two contributions: the mobile contribution derived above and the injection contribution derived in §2. In that section, it was proved that for the present configuration of a dislocation and its image, the injection contributions vanish at the free surface (*x*=*l*) as required. Hence, it only remains to prove that the mobile contributions also vanish.

Considering the image dislocation's field in the local coordinate system:

3.7 $$\sigma_{xy}^{\mathrm{im},\mathrm{mob}}(x^{\prime }=l,z^{\prime },t)=\frac{\mu B}{2\pi }\frac{\partial }{\partial t}\int_{0}^{\infty }H(\tilde{t}-{\tilde{r}}^{\prime }b)\frac{lz^{\prime }(b^{2}{\tilde{r}}^{\prime 2}-2{\tilde{t}}^{2})}{{\tilde{r}}^{\prime 4}T_{b}}\;\text{d}\xi$$

and the dislocation's

3.8 $$\sigma_{xy}^{\mathrm{dis},\mathrm{mob}}(x=l,z,t)=\frac{\mu B}{2\pi }\frac{\partial }{\partial t}\int_{0}^{\infty }H(\tilde{t}-\tilde{r}b)\frac{lz(b^{2}{\tilde{r}}^{2}-2{\tilde{t}}^{2})}{{\tilde{r}}^{4}T_{b}}\;\text{d}\xi$$

and transforming coordinates for the image dislocation (i.e. inverting the sign of *z*′) and summing both contributions,

3.9 $$\begin{array}{rl} \sigma_{xy}^{\mathrm{tot},\mathrm{mob}}(x=l,z,t) & =\frac{\mu B}{2\pi }\frac{\partial }{\partial t}\int_{0}^{\infty }H(\tilde{t}-\tilde{r}b)\frac{l(-z)(b^{2}{\tilde{r}}^{2}-2{\tilde{t}}^{2})}{{\tilde{r}}^{4}T_{b}}\;\text{d}\xi \\ & \;+\frac{\mu B}{\pi }\frac{\partial }{\partial t}\int_{0}^{\infty }H(\tilde{t}-\tilde{r}b)\frac{lz(b^{2}{\tilde{r}}^{2}-2{\tilde{t}}^{2})}{{\tilde{r}}^{4}T_{b}}\;\text{d}\xi . \end{array}$$

It is clear that the first integral is the negative of the second, so the sum vanishes, as expected.

The image force is then given by $F_{x}(t)=B\sigma_{xy}^{\mathrm{im},\mathrm{mob}+\mathrm{inj}}(x^{\prime }=2l,z^{\prime }=0,t)$, which in this case is formed by,

3.10 $$\begin{array}{rl} F_{x}(t) & =\frac{\mu B^{2}}{2\pi }\frac{\partial }{\partial t}\int_{0}^{\infty }H(t-\eta (\xi )-(2l-\xi )b)\frac{\sqrt{{\left(t-\eta (\xi )\right)}^{2}-b^{2}{\left(2l-\xi \right)}^{2}}}{{\left(2l-\xi \right)}^{2}}\;\text{d}\xi \\ & \;+\frac{\mu B^{2}}{2\pi }\frac{t\sqrt{t^{2}-4b^{2}l^{2}}}{4b^{2}l^{3}-2lt^{2}}H(t-2bl). \end{array}$$

It is worth noticing that in this case the order of integration and differentiation can be exchanged because all singularities are integrable, whereby

3.11 $$\begin{array}{rl} F_{x}(t) & =\frac{\mu B^{2}}{2\pi }\int_{0}^{\infty }H(t-\eta (\xi )-(2l-\xi )b)\frac{t-\eta (\xi )}{{\left(2l-\xi \right)}^{2}\sqrt{(t-\eta {\left(\xi )\right)}^{2}-b^{2}{\left(2l-\xi \right)}^{2}}}\;\text{d}\xi \\ & \;+\frac{\mu B^{2}}{2\pi }\frac{t\sqrt{t^{2}-4b^{2}l^{2}}}{4b^{2}l^{3}-2lt^{2}}H(t-2bl). \end{array}$$

This is the general form of the image force experienced by a non-uniformly moving injected screw dislocation, moving towards the surface with a past history prescribed by *t*=*η*(*ξ*).

The case of the uniformly moving dislocation can be more revealing. Following the same procedure, one finds that when *η*(*ξ*)=*d*⋅*ξ*, where *d*=1/*v* is the dislocation's uniform slowness,

3.12 $$F_{x}(t)=\frac{\mu B^{2}}{2\pi }\frac{\sqrt{t^{2}-4b^{2}l^{2}}(b^{2}l-dt)}{\left(t^{2}-2b^{2}l^{2})(t-2dl\right)}H(t-2bl).$$

**Figure 3.**
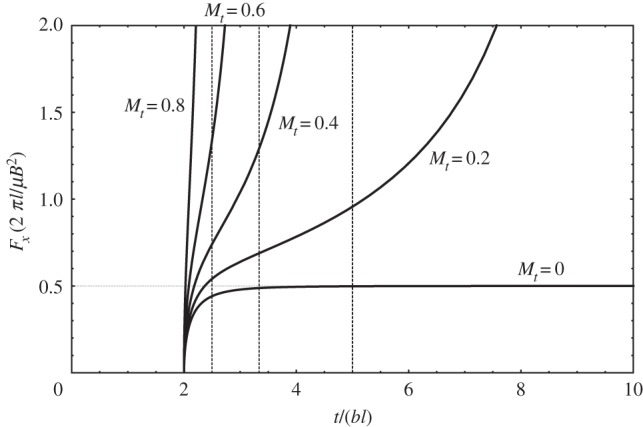
Evolution of the time-dependent image force's magnitude (non-dimensional) with time. The dashed vertical lines represent the instant in time when the dislocation reaches the free surface; for *M*_*t*_=0.2, the line is not represented, as the dislocation reaches the free surface when *t*=10*bl*.

Figure 3 shows the magnitude of the image force for dislocations moving towards the surface at different uniform speeds, which in the figure are represented via *M*_*t*_=*v*/*c*_*t*_, the transverse Mach number. The quiescent, injected dislocation's case is recovered when *M*_*t*_=0. Otherwise, as can be seen in figure 3, the magnitude of the image force is seen to increase with increasing speed and time, suggesting that once the retardation entailed by the finite propagation of the elastodynamic fields is overcome, the time-dependent image force is of larger magnitude than the corresponding quiescent dislocation's.

Necessarily, in this framework the dislocation will reach the surface; this occurs when *t*=2*dl*; at that instant of time, the image force in equation (3.12) diverges (note the (*t*−2*dl*) term in the denominator). In figure 3, this is signalled with vertical dashed lines. Also necessarily, a dislocation moving at the transverse speed of sound (i.e. *M*_*t*_=1) cannot experience an image force: it will reach the surface at the same time as its elastic fields.

More importantly, however, the fact that the magnitude of the image force increases with time for any one uniform speed suggests that the image force should in fact accelerate the dislocation towards the surface. This is because, in the absence of additional dissipative mechanisms, the image force will attract the dislocation towards the surface with increasing magnitude, which should translate in an increase in the speed of the dislocation as it approaches the surface. This effect is different from the increase in the magnitude of the image force that one sees in the elastostatic (and quiescent injected) case; the latter is also captured here, and is the result of the decrease in the distance *l* (note the 1/*l* scaling factor in the vertical axis in figure 3). The difference can be best seen by rewriting equation (3.12) as follows:

3.13 $$F_{x}(t)=\frac{\mu B^{2}}{2\pi }\left[\frac{1}{l}\right]\underset{\mathrm{Dynamic}\;\mathrm{contribution}}{\underbrace{\left[\frac{(M_{t}-\tau )\sqrt{\tau^{2}-4}}{\left(2-M_{t}\tau )(\tau^{2}-2\right)}\right]H(\tau -2)}},$$

where *τ*=*t*/(*bl*). Clearly, the 1/*l* term in the first bracket corresponds with the prediction provided by the elastostatic image force; the term in the second bracket, dependent on the speed of the dislocation, is a dynamic contribution to the image force which multiplies the elastostatic contribution. As discussed above, for any *M*_*t*_>0 this contribution invariably makes the image force larger as time advances, and increases in magnitude with increasing speed. Thus, as a result of their dynamic, time-dependent fields, moving screw dislocations will be subjected to an image force stronger than that predicted by elastostatics, once the retardation time is overcome.

The significance of the dynamic contribution to the magnitude of the image force is great, even at very low dislocation speeds. For instance, for *M*_*t*_=0.05, corresponding to dislocation speeds of about ≈100−200 ms^−1^ for most metals, the dynamic contribution doubles the magnitude of the image force with respect to that of the elastostatic prediction's at time *t*≈10*bl*; for systems of the order of magnitude of micrometres, this timescale is of the order of 1−5 *ns*. Thus, due to elastodynamic effects, the magnitude of the image force doubles with respect to the elastostatic prediction even for very low dislocation speeds, and well within the timescale of a quasi-static discrete dislocation dynamics simulation (cf. [3]). This suggests that even in quasi-static applications of plasticity, dislocations will tend towards free surfaces at a much faster rate than predicted by elastostatics.

## 4. General formulation of the elastodynamic image field componentsof an edge dislocation

The fields of an edge dislocation were derived, for the injected case, by Gurrutxaga-Lerma *et al.* [3], and for the mobile case by Markenscoff & Clifton [5]. The solution procedure mirrors that described in the previous section for the screw dislocation. In this section, a general procedure is given for the derivation of the elastodynamic image forces of a straight edge dislocation in the presence of a free surface, both for a quiescent, injected edge dislocation and for an injected, moving edge dislocation.

As in the well-known elastostatic case (cf. [1]), the problem of edge dislocations in the presence of a free surface cannot simply be solved, as done in the screw case, by employing the image dislocation construction. This is because the *σ*_*xz*_ shear stress components of the dislocation and its image do not vanish on the surface. Hence, further considerations are necessary before an expression of the image forces acting on an edge dislocation can be computed.

The general problem to solve is be the following:

(a) **Governing equations**:
  4.1 $$\frac{\partial^{2}\phi }{\partial x^{2}}+\frac{\partial^{2}\phi }{\partial z^{2}}=a^{2}\frac{\partial^{2}\phi }{\partial t^{2}}$$
  and
  4.2 $$\frac{\partial^{2}\psi }{\partial x^{2}}+\frac{\partial^{2}\psi }{\partial z^{2}}=b^{2}\frac{\partial^{2}\psi }{\partial t^{2}},$$
  where *a*=1/*c*_*l*_ is the longitudinal slowness of sound, and where *ϕ* and *ψ* are the Kelving–Helmholtz potentials, defined such that
  4.3 $$\begin{array}{rl} u_{x} & =\frac{\partial \phi }{\partial x}-\frac{\partial \psi }{\partial z}, \end{array}$$
  4.4 $$\begin{array}{rl} u_{z} & =\frac{\partial \phi }{\partial z}+\frac{\partial \psi }{\partial x}, \end{array}$$
  4.5 $$\begin{array}{rl} \sigma_{xx} & =\Lambda \left(\frac{\partial^{2}\phi }{\partial x^{2}}+\frac{\partial^{2}\phi }{\partial z^{2}}\right)+2\mu \left(\frac{\partial^{2}\phi }{\partial x^{2}}-\frac{\partial^{2}\psi }{\partial x\partial z}\right) \end{array}$$
  4.6 $$\begin{array}{rl} \text{and}\;\;\sigma_{xz} & =\mu \left(2\frac{\partial^{2}\phi }{\partial x\partial z}+\frac{\partial^{2}\psi }{\partial x^{2}}-\frac{\partial^{2}\psi }{\partial z^{2}}\right). \end{array}$$
(b) **Boundary conditions**:
  4.7 $$\begin{array}{rl} u_{x}(x,0,t) & =BH(-x)H(t)\;\text{or}\;u_{x}(x,0,t)=BH(l(t)-x)H(t), \end{array}$$
  4.8 $$\begin{array}{rl} \sigma_{zz}(x,0,t) & =0 \end{array}$$
  4.9 $$\begin{array}{rl} \text{and}\;\;\sigma_{xz}(l,z,t) & =0\;\forall z,t\in R. \end{array}$$

The displacement boundary condition models the dislocation as a Volterra discontinuity with Burgers vector parallel to the positive *x*-axis; the *σ*_*zz*_ boundary condition ensures that the dislocation causes no normal stress anywhere on the slip plane. The *σ*_*xz*_ boundary condition (equation (4.9)) corresponds with the free surface requirement. It is important to note that it applies for any value of *z*, while the rest of boundary conditions apply for any value of *x*.

The usual solution method (see [2,3,5]) involves the successive use of a Laplace transform in time and a bilateral Laplace transform (or Fourier transform) in the spatial variable (in the case of a dislocation injected in an infinite medium, *x*). In this case, this is not possible because *σ*_*xz*_ is applied ∀*z* and *u*_*x*_, ∀*x*.

Still, the problem can be solved invoking the image dislocation construction, in a manner akin to how it is done for the elastostatic case. As shown in figure 1*c*,*d*, the image construction considers an image edge dislocation of opposite sign but the same magnitude located at a distance *l* from the surface.

It can be shown that, as in the elastostatic case, due to symmetry about *x*, all stress components acting on the surface vanish except for *σ*_*xz*_ [3]. The distribution of *σ*_*xz*_(*z*,*t*) at the free surface can be obtained by superimposing the contributions due to the image and the actual dislocations. For brevity, this stress distribution at the free surface will be called *σ*_*xz*_(*l*,*z*,*t*)=*ζ*(*z*,*t*).

Thus, one needs to solve *Lamb's problem* for the following boundary conditions:

4.10 $$\sigma_{xz}(0^{+},z,t)=\zeta (z,t)\;\mathrm{and}\;\sigma_{xx}(0^{+},z,t)=0,$$

where the following translation in *x* has been used for simplicity:

x↦x−l.

The same successive Laplace transforms in time and bilateral Laplace transform in space defined in equations (2.4) and (2.5) are used here. Apply them successively to the governing equations to obtain

4.11 $$\frac{\partial^{2}\Phi }{\partial x^{2}}=\alpha^{2}s^{2}\Phi ,$$

where *α*^2^=*a*^2^−λ^2^ and

4.12 $$\frac{\partial^{2}\Psi }{\partial x^{2}}=\beta^{2}s^{2}\Psi ,$$

where *β*^2^=*b*^2^−λ^2^.

The solution to these equations are

4.13 $$\Phi (x,\lambda ,s)=C_{\phi }(\lambda ,s)\;e^{-s\alpha x}$$

and

4.14 $$\Psi (x,\lambda ,s)=C_{\psi }(\lambda ,s)\;e^{-s\beta x}.$$

Here, *C*_*ϕ*_(λ,*s*) and *C*_*ψ*_(λ,*s*) are integration constants to be found by applying the boundary conditions.

Applying the transforms to the boundary conditions (equation (4.10)), one obtains

4.15 $$\Sigma_{xz}(0^{+},\lambda ,s)=Z(\lambda ,s)\;\mathrm{and}\;\Sigma_{xx}(0^{+},\lambda ,s)=0,$$

where $Z(\lambda ,s)=L_{x}\{L_{t}\{\zeta (z,t)\}\}$.

Recall that

$$\sigma_{xz}=\mu \left(2\frac{\partial^{2}\phi }{\partial x\partial z}+\frac{\partial^{2}\psi }{\partial x^{2}}-\frac{\partial^{2}\psi }{\partial z^{2}}\right)\;\text{and}\;\sigma_{xx}=\Lambda \left(\frac{\partial^{2}\phi }{\partial x^{2}}+\frac{\partial^{2}\phi }{\partial z^{2}}\right)+2\mu \left(\frac{\partial^{2}\phi }{\partial x^{2}}-\frac{\partial^{2}\psi }{\partial x\partial z}\right).$$

In transformed space, they become

4.16 $$\Sigma_{xz}=\mu \left(2s\lambda \frac{\partial \Phi }{\partial x}+\frac{\partial^{2}\Psi }{\partial x^{2}}-s^{2}\lambda^{2}\Psi \right)$$

and

4.17 $$\Sigma_{xx}=\Lambda \left(\frac{\partial^{2}\Phi }{\partial x^{2}}+\lambda^{2}s^{2}\Phi \right)+2\mu \left(\frac{\partial^{2}\Phi }{\partial x^{2}}-\lambda s\frac{\partial \Psi }{\partial x}\right).$$

Substituting equations (4.13) and (4.14) in equations (4.16) and (4.17), then equating this to the boundary conditions (equation (4.15)) for *x*=0^+^, and using the fact that (*Λ*+2*μ*)/*μ*=*b*^2^/*a*^2^, one obtains the following system of equations, with *C*_*ϕ*_(λ,*s*) and *C*_*ψ*_(λ,*s*) as unknowns:

4.18 $$\left[\begin{array}{cc} -2\mu \lambda \alpha s^{2} & \mu (b^{2}-2\lambda^{2})s^{2}\\ \mu (b^{2}-2\lambda^{2})s^{2} & 2\mu \lambda \beta s^{2} \end{array}\right]\left[\begin{array}{c} C_{\phi }(\lambda ,s)\\ C_{\psi }(\lambda ,s) \end{array}\right]=\left[\begin{array}{c} Z(\lambda ,s)\\ 0 \end{array}\right],$$

the solution to which is

4.19 $$C_{\phi }(\lambda ,s)=\frac{1}{s^{2}\mu R(\lambda )}[-2\lambda \alpha ]Z(\lambda ,s)$$

and

4.20 $$C_{\psi }(\lambda ,s)=\frac{1}{s^{2}\mu R(\lambda )}\left[2\lambda^{2}-b^{2}\right]Z(\lambda ,s),$$

where

4.21 $$R(\lambda )=-4\alpha \beta \lambda^{2}-{\left(b^{2}-2\lambda^{2}\right)}^{2}$$

is the *Rayleigh function*.

It follows that

4.22 $$\Phi (x,\lambda ,s)=\frac{1}{s^{2}\mu R(\lambda )}[-2\lambda \alpha ]Z(\lambda ,s)\;e^{-s\alpha x}$$

and

4.23 $$\Psi (x,\lambda ,s)=\frac{1}{s^{2}\mu R(\lambda )}\left[2\lambda^{2}-b^{2}\right]Z(\lambda ,s)\;e^{-s\beta x}.$$

It is clear that the form of *C*_*ϕ*_ and *C*_*ψ*_ is heavily dependent on the form of *Z*(λ,*s*). An inspection of *ζ*(*z*,*t*) (see equations (5.1) and (6.2)) suggests that for the current case, its Laplace transforms in time alone will be exceedingly complex, taking the form of Bessel and Struve functions in *s* (cf. [6]).

An alternative approach can, however, be considered. The stress component relevant to the derivation of the image force on an edge dislocation is *σ*_*xz*_. Substituting equations (4.11) and (4.12) in equation (4.16), one finds

4.24 $$\Sigma_{xz}(x,\lambda ,s)=\Gamma (x,\lambda ,s)Z(\lambda ,s),$$

where

$$\Gamma (x,\lambda ,s)=\frac{1}{R(\lambda )}[4\lambda^{2}\alpha^{2}\;e^{-s\alpha x}-{\left(b^{2}-2\lambda^{2}\right)}^{2}\;e^{-s\beta x}].$$

From equation (4.24), it is clear that the desired stress component is expressed as the product of two Laplace transforms, whereby the stress component *σ*_*xz*_ in the cartesian space (*x*,*z*,*t*) must be the double convolution of the untransformed functions,

4.25 $$\sigma_{xz}(x,z,t)=\int_{-\infty }^{\infty }\int_{0}^{\infty }[G(x,\varsigma -z,\vartheta -t)\zeta (\varsigma ,\vartheta )]\;\text{d}\vartheta \;\text{d}\varsigma ,$$

where $G(x,z,t)=L_{t}^{-1}\{L_{z}^{-1}\{\Gamma (x,\lambda ,s)\}\}$. It is worth noticing that *G*(*x*,*z*,*t*) is in fact the first derivative of the system's elastodynamic half-space Green's tensor.

All that remains is therefore to invert *Γ*(*x*,λ,*s*), which can be achieved applying the Cagniard–de Hoop method. For simplicity, split *Γ*(*x*,λ,*s*) into two terms, *Γ*=*Γ*_*a*_+*Γ*_*b*_, where

4.26 $$\Gamma_{a}(x,\lambda ,s)=\frac{4\lambda^{2}\alpha^{2}}{R(\lambda )}\;e^{-s\alpha x}\;\text{and}\;\Gamma_{b}(x,\lambda ,s)=\frac{-{\left(b^{2}-2\lambda^{2}\right)}^{2}}{R(\lambda )}\;e^{-s\beta x}.$$

The inversion procedure is analogous for both. Consider *Γ*_*a*_,

4.27 $${\hat{G}}_{a}(x,z,s)=\frac{1}{2\pi i}\int_{-i\infty }^{i\infty }\left[\frac{4\lambda^{2}\alpha^{2}}{R(\lambda )}\right]e^{-s(\alpha x-\lambda z)}s\;\text{d}\tau .$$

The Cagniard path will be given by *τ*=*αx*−λ*z*, whereby

4.28 $$\begin{array}{rlr} \lambda & =\frac{-\tau z\pm ix\sqrt{\tau^{2}-(x^{2}+z^{2})a^{2}}}{x^{2}+z^{2}},\;\alpha =\frac{\tau x\pm iz\sqrt{\tau^{2}-(x^{2}+z^{2})a^{2}}}{x^{2}+z^{2}}\;\text{and}\;\beta & =\sqrt{b^{2}-\lambda^{2}}. \end{array}$$

Following the Cagniard–de Hoop method, the path is distorted as done in §3. The positive branch (λ>0) is chosen, leading to analogous results. It is worth noticing that *R*(λ) has roots for λ_*R*_=1/*c*_*R*_, where *c*_*R*_ is the Rayleigh wave speed; however, λ_*R*_>*b*, so the branch cut defined by $\left(-\infty ,-\lambda_{R}]\cup [+\lambda_{R},\infty \right)$ is contained in both (−∞,−b]∪[+b,∞) and (−∞,−a]∪[+a,∞).

Thus, define

4.29 $$F_{a}(\tau )=\text{Im}\left[\frac{4\lambda_{+}^{2}\alpha_{+}^{2}}{R(\lambda_{+})}\frac{\partial \lambda_{+}}{\partial \tau }\right].$$

Then, it is found that

4.30 $${\hat{G}}_{a}(x,z,s)=\frac{s}{\pi }\int_{ra}^{\infty }F_{a}(\tau )\;e^{-s\tau }\;\text{d}\tau =\frac{s}{\pi }\int_{0}^{\infty }H(\tau -ra)F_{a}(\tau )\;e^{-s\tau }\;\text{d}\tau ,$$

so that the fully inverted function will be

4.31 $$G_{a}(x,z,t)=\frac{1}{\pi }H(t-ra)\frac{\text{d}F_{a}(t)}{\text{d}t}.$$

Repeating the same process for *G*_*b*_, one would define the Cagniard path as *τ*=*βx*−λ*z*, and accordingly define

4.32 $$\begin{array}{rl} \lambda & =\frac{-\tau z\pm ix\sqrt{\tau^{2}-(x^{2}+z^{2})a^{2}}}{x^{2}+z^{2}},\;\beta =\frac{\tau x\pm iz\sqrt{\tau^{2}-(x^{2}+z^{2})b^{2}}}{x^{2}+z^{2}}\;\text{and}\;\alpha =\sqrt{a^{2}-\lambda^{2}}. \end{array}$$

In this case, it is found that

4.33 $$F_{b}(\tau )=\text{Im}\left[\frac{-{\left(b^{2}-2\lambda_{+}^{2}\right)}^{2}}{R(\lambda_{+})}\frac{\partial \lambda_{+}}{\partial \tau }\right].$$

Following the same procedure as above, one would finally be able to construct *G*(*x*,*z*,*t*) as

4.34 $$G(x,z,t)=\frac{1}{\pi }\left[H(t-ra)\frac{\text{d}F_{a}(t)}{\text{d}t}+H(t-rb)\frac{\text{d}F_{b}(t)}{\text{d}t}\right].$$

The explicit form of both *F*_*a*_(*t*) and *F*_*b*_(*t*) is somewhat lengthy and can be written as

4.35 $$F_{a}(t)=\frac{N_{a}}{D_{a}}\;\text{and}\;F_{b}(t)=\frac{N_{b}}{D_{b}}$$

after defining

4.36 $$\begin{array}{rl} N_{a} & =8\{tx[T_{a}z(b^{4}r^{8}(t^{2}+T_{a}^{2})(x^{2}-z^{2})+4b^{2}r^{4}{\left(t^{2}z^{2}+T_{a}^{2}x^{2}\right)}^{2}\\ & \;-4(t^{2}-T_{a}^{2})(x^{2}+z^{2}){\left(t^{2}z^{2}+T_{a}^{2}x^{2}\right)}^{2})+2r^{2}\rho_{a}\sin(\theta_{a})(t^{2}x^{2}+T_{a}^{2}z^{2}){\left(t^{2}z^{2}+T_{a}^{2}x^{2}\right)}^{2}]\\ & \;-2r^{2}\rho_{a}T_{a}z\cos(\theta_{a}){\left(t^{2}z^{2}+T_{a}^{2}x^{2}\right)}^{2}(t^{2}x^{2}+T_{a}^{2}z^{2})\}, \end{array}$$

4.37 Da=r16{b8+1r48b6(Tax−tz)(tz+Tax)+1r8[8b4(r2ρa(Tazsin⁡(θa)(x2(2t2+Ta2)−t2z2)+txcos⁡(θa)(z2(t2+2Ta2)−Ta2x2))+3t4z4−2t2Ta2x2z2+3Ta4x4)]+1r12[32b2(t2z2+Ta2x2)2(r2ρa(Tazsin⁡(θa)−txcos⁡(θa))+(tz+Tax)(Tax−tz))]+1r16[16(t2z2+Ta2x2)2(r4ρa2(t2x2+Ta2z2)+2r2ρa(Tazsin⁡(θa)(Ta2x2−t2(2x2+z2))1r4+txcos⁡(θa)(t2z2−Ta2(x2+2z2)))+(t2z2+Ta2x2)2)]},

4.38 $$\begin{array}{rl} N_{b} & =tx\{-b^{8}r^{16}-16b^{6}r^{12}T_{b}^{2}z^{2}+8b^{4}r^{8}(t^{4}z^{4}+10t^{2}T_{b}^{2}x^{2}z^{2}+T_{b}^{4}x^{4})\\ & \;-8r^{2}\rho_{b}T_{b}z\sin(\theta_{b})(t^{2}x^{2}+T_{b}^{2}z^{2})(b^{4}r^{8}-4{\left(t^{2}z^{2}+T_{b}^{2}x^{2}\right)}^{2})+64b^{2}r^{4}{\left(t^{2}T_{b}z^{3}+T_{b}^{3}x^{2}z\right)}^{2}\\ & \;-16{\left(t^{2}z^{2}+T_{b}^{2}x^{2}\right)}^{4}\}-4r^{2}\rho_{b}\cos(\theta_{b})(t^{2}x^{2}+T_{b}^{2}z^{2})[b^{4}r^{8}(t^{2}z^{2}-T_{b}^{2}x^{2})\\ & \;+4b^{2}r^{4}{\left(t^{2}z^{2}+T_{b}^{2}x^{2}\right)}^{2}-4(T_{b}^{2}x^{2}-t^{2}z^{2}){\left(t^{2}z^{2}+T_{b}^{2}x^{2}\right)}^{2}] \end{array}$$

4.39 $$\begin{array}{rl} \text{and}\;\;D_{b} & =r^{2}T_{b}\{b^{8}r^{16}+8b^{6}r^{12}(tz+T_{b}x)(T_{b}x-tz)+8b^{4}r^{8}(3t^{4}z^{4}-2t^{2}T_{b}^{2}x^{2}z^{2}+3T_{b}^{4}x^{4})\\ & \;+32b^{2}r^{4}(tz+T_{b}x)(T_{b}x-tz){\left(t^{2}z^{2}+T_{b}^{2}x^{2}\right)}^{2}\\ & \;+8r^{2}\rho_{b}[T_{b}z\sin(\theta_{b})(b^{4}r^{8}(x^{2}(2t^{2}+T_{b}^{2})-t^{2}z^{2})\\ & \;+4b^{2}r^{4}{\left(t^{2}z^{2}+T_{b}^{2}x^{2}\right)}^{2}-4{\left(t^{2}z^{2}+T_{b}^{2}x^{2}\right)}^{2}(t^{2}(2x^{2}+z^{2})-T_{b}^{2}x^{2}))\\ & \;+tx\cos(\theta_{b})(b^{4}r^{8}(z^{2}(t^{2}+2T_{b}^{2})-T_{b}^{2}x^{2})-4b^{2}r^{4}{\left(t^{2}z^{2}+T_{b}^{2}x^{2}\right)}^{2}\\ & \;-4{\left(t^{2}z^{2}+T_{b}^{2}x^{2}\right)}^{2}(T_{b}^{2}(x^{2}+2z^{2})-t^{2}z^{2}))]\\ & \;+16{\left(t^{2}z^{2}+T_{b}^{2}x^{2}\right)}^{2}(r^{4}\rho_{b}^{2}(t^{2}x^{2}+T_{b}^{2}z^{2})+{\left(t^{2}z^{2}+T_{b}^{2}x^{2}\right)}^{2})\}, \end{array}$$

where $T_{a}=\sqrt{t^{2}-a^{2}r^{2}}$, $T_{b}=\sqrt{t^{2}-b^{2}r^{2}}$, and

4.40 $$\begin{array}{rl} \rho_{a}(x,z,t) & ={\left[\frac{t^{4}-2t^{2}(a^{2}x^{2}+b^{2}(z^{2}-x^{2}))+{\left(a^{2}x^{2}-b^{2}(x^{2}+z^{2})\right)}^{2}}{{\left(x^{2}+z^{2}\right)}^{2}}\right]}^{1/4}, \end{array}$$

4.41 $$\begin{array}{rl} \tan[2\theta_{a}(x,z,t)] & =\frac{2txz\sqrt{t^{2}-a^{2}(x^{2}+z^{2})}}{t^{2}(x^{2}-z^{2})-(x^{2}+z^{2})(a^{2}x^{2}-b^{2}(x^{2}+z^{2}))}, \end{array}$$

4.42 $$\begin{array}{rl} \rho_{b}(x,z,t) & ={\left[\frac{t^{4}-2t^{2}(b^{2}x^{2}+a^{2}(z^{2}-x^{2}))+(b^{2}x^{2}-a^{2}{\left(x^{2}+z^{2})\right)}^{2}}{{\left(x^{2}+z^{2}\right)}^{2}}\right]}^{1/4} \end{array}$$

4.43 $$\begin{array}{rl} \text{and}\;\;\tan[2\theta_{b}(x,z,t)] & =\frac{2txz\sqrt{t^{2}-b^{2}(x^{2}+z^{2})}}{t^{2}(x^{2}-z^{2})-(x^{2}+z^{2})(b^{2}x^{2}-a^{2}(x^{2}+z^{2})).} \end{array}$$

Having found *F*_*a*_ and *F*_*b*_ one can proceed to compute their time derivatives (not reproduced here due to their length, but reproduced in the electronic supplementary material) and construct *G*(*x*,*z*,*t*) as defined in equation (4.34). One can then obtain the image field as the convolution described in equation (4.25). Of interest here is to note that *G*(*x*,*z*,*t*) is antisymmetric (odd) with respect to *z*.

## 5. Image force on an injected, quiescent edge dislocation

For the case of an injected, quiescent dislocation, the *σ*_*xz*_(*z*,*t*)=*ζ*(*z*,*t*) distribution at the free surface can be calculated to be [3]

5.1 $$\begin{array}{rl} \sigma_{xz}(l,z,t) & =\zeta (z,t)=-\frac{\mu B}{2\pi b^{2}}\{4\frac{2lt(a^{2}(-l^{4}+l^{2}z^{2}+2z^{4})+t^{2}(l^{2}-3z^{2}))}{{\left(l^{2}+z^{2}\right)}^{3}\sqrt{t^{2}-a^{2}(l^{2}+z^{2})}}H(t-a\sqrt{l^{2}+z^{2}})\\ & \;+\frac{2lt(b^{4}(-l^{6}-7l^{4}z^{2}+l^{2}z^{4}+7z^{6})+4b^{2}t^{2}(l^{4}-5z^{4})-4t^{4}(l^{2}-3z^{2}))}{{\left(l^{2}+z^{2}\right)}^{3}(t^{2}-b^{2}z^{2})\sqrt{t^{2}-b^{2}(l^{2}+z^{2})}}H(t-b\sqrt{l^{2}+z^{2}})\}. \end{array}$$

The problem can be slightly simplified by noting that the dislocation core lies along the epicentral *x*-axis, so equation (4.25) needs be solved for *z*=0 alone. Thus, one needs to find

5.2 $$\sigma_{xz}(-l,0,t)=\int_{-\infty }^{\infty }\int_{0}^{\infty }[G(-l,\varsigma ,\vartheta -t)\zeta (\varsigma ,\vartheta )]\;\text{d}\vartheta \;\text{d}\varsigma .$$

The value of this double convolution is zero for *σ*_*xz*_(−*l*,0,*t*). This is in direct analogy with the elastostatic case (cf. [1]). One can check this is true by direct integration of the double convolution.

Alternatively, one can convince oneself of the veracity of this assertion by invoking the symmetries in both *G*(*x*,*z*,*t*) and *ζ*(*z*,*t*). For simplicity, rewrite equation (5.2) as follows:

5.3 $$\begin{array}{rl} \sigma_{xz}(-l,0,t) & =\int_{R}[G(-l,\varsigma ,\vartheta -t)\zeta (\varsigma ,\vartheta )]\;\text{d}\vartheta \;\text{d}\varsigma =\int_{R}\zeta_{a}(\varsigma ,\vartheta )G_{a}(-l,\varsigma ,\vartheta -t)\;\text{d}\vartheta \;\text{d}\varsigma \\ & \;+\int_{R}[G_{a}(-l,\varsigma ,\vartheta -t)\zeta_{b}(\varsigma ,\vartheta )+G_{b}(-l,\varsigma ,\vartheta -t)\zeta_{a}(\varsigma ,\vartheta )]\;\text{d}\vartheta \;\text{d}\varsigma \\ & \;+\int_{R}G_{b}(-l,\varsigma ,\vartheta -t)\zeta_{b}(\varsigma ,\vartheta )\;\text{d}\vartheta \;\text{d}\varsigma , \end{array}$$

where R≡(−∞,∞)×[0,∞) and

5.4 $$\zeta_{a}(\varsigma ,\vartheta )=-\frac{\mu B}{2\pi b^{2}}\frac{8l\vartheta (a^{2}(-l^{4}+l^{2}\varsigma^{2}+2\varsigma^{4})+\vartheta^{2}(l^{2}-3\varsigma^{2}))}{{\left(l^{2}+\varsigma^{2}\right)}^{3}\sqrt{\vartheta^{2}-a^{2}(l^{2}+\varsigma^{2})}}H(\vartheta -a\sqrt{l^{2}+\varsigma^{2}})$$

and

5.5 $$\begin{array}{rl} \zeta_{b}(\varsigma ,\vartheta ) & =-\frac{\mu B}{2\pi b^{2}}\frac{2l\vartheta (b^{4}(-l^{6}-7l^{4}\varsigma^{2}+l^{2}\varsigma^{4}+7\varsigma^{6})+4b^{2}\vartheta^{2}(l^{4}-5\varsigma^{4})-4\vartheta^{4}(l^{2}-3\varsigma^{2}))}{{\left(l^{2}+\varsigma^{2}\right)}^{3}(\vartheta^{2}-b^{2}\varsigma^{2})\sqrt{\vartheta^{2}-b^{2}(l^{2}+\varsigma^{2})}}\\ & \;\times H(\vartheta -b\sqrt{l^{2}+\varsigma^{2}}), \end{array}$$

so that *ζ*(*ς*,*ϑ*)=*ζ*_*a*_(*ς*,*ϑ*)+*ζ*_*b*_(*ς*,*ϑ*).

As remarked in §4, both *G*_*a*_(−*l*,*ς*,*ϑ*−*t*) and *G*_*b*_(−*l*,*ς*,*ϑ*−*t*) are odd functions with respect to *ς*, and both *ζ*_*a*_(*ς*,*ϑ*) and *ζ*_*b*_(*ς*,*ϑ*) are even functions with respect to *ς*. Hence, the Cauchy principal values of the convolution integral with respect to *ς* must vanish and so must, it follows, the whole convolution integral. It must be stressed that this is only true in this case because the convolution is being evaluated along the epicentral line (*z*=0); for any other value of *z*, the convolution need not generally vanish.

As the convolution integrals vanish, the term due to the free surface's boundary condition also vanishes. This situation mirrors that occurring in the elastostatic case, where the image force terms due to the free surface are proved to vanish for the dislocation [1].

Accordingly, the image force on an injected, quiescent edge dislocation located at a distance *l* from a flat-free surface can be obtained from the image dislocation's *σ*_*xz*_ stress field alone, evaluated at the position of the actual dislocation's core in the same manner it was done for the screw dislocation's case. It is found to be given by

5.6 $$F_{x}(t)=\frac{\mu B^{2}}{2\pi b^{2}}\left[-\frac{t\sqrt{t^{2}-4a^{2}l^{2}}}{2\;l^{3}}H(t-2al)-\frac{{\left(t^{2}-2b^{2}l^{2}\right)}^{2}}{2l^{3}t\sqrt{t^{2}-4b^{2}l^{2}}}H(t-2bl)\right].$$

In order to allow easy comparison with the elastostatic image force (equation (1.1)), this equation can be rewritten as

5.7 $$F_{x}(\kappa )=\frac{\mu B^{2}}{2\pi (1-\nu )}\left[\frac{1}{l}\right]\underset{\mathrm{Dynamic}\;\mathrm{term}}{\underbrace{\left[\frac{1}{2(1-R^{2})}\left(\frac{R^{2}\kappa \sqrt{\kappa^{2}-4}}{2}H(\kappa -2)-\frac{{\left(R^{2}\kappa^{2}-2\right)}^{2}}{2R\kappa \sqrt{R^{2}\kappa^{2}-4}}H\left(\kappa -\frac{2}{R}\right)\right)\right]}},$$

where in this case *κ*=*t*/*al* and *R*=*a*/*b*.

**Figure 4.**
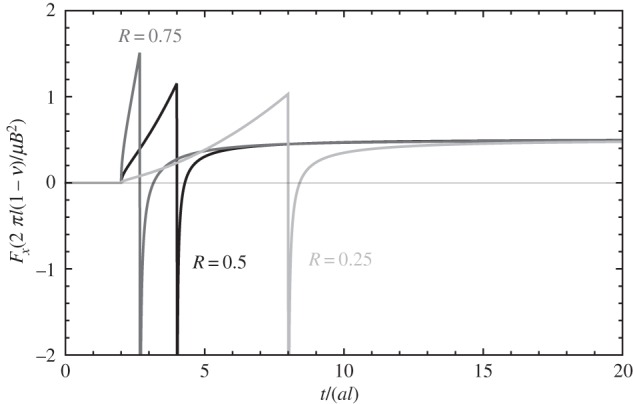
Elastodynamic image force on an injected, quiescent edge dislocation, for different *R*=*a*/*b* ratios. Note that the values $R\in [0,\sqrt{3}/2]$ because $R=\sqrt{\left(1-2\nu )/(2(1-\nu )\right)}$, so the value of this ratio is limited by the value that Poisson's ratio *ν* can take, *ν*∈[−1,0.5].

The magnitude of the image force, *F*_*x*_, is represented in figure 4. As can be seen both in figure 4 and equation (5.7), the image force consists of two separate wavefronts: one corresponding to the longitudinal wave, which arrives at the dislocation at *t*=2*al*, and a second one corresponding to the transverse wave, which arrives at the dislocation at *t*=2*bl*=2*al*/*R*. Both arrival times are the retardation times incurred by the initial longitudinal and transverse wavelets that are emitted from the injected dislocation's core, reach the surface at *t*=*al* and *t*=*bl*, respectively, and are reflected back to the core. The arrival of the transverse wavefront is marked by a 1/*t* singularity, which is a feature of the fields of an injected edge dislocation [3].

As can be seen in figure 4, right after the arrival of the transverse wavefront at *t*=2*bl*, the image force experiences a brief, transient reversal of its sign—for the duration of this transient event the image force becomes repulsive. This reversal has finite duration that depends solely on the material's elastic constants: the reversal begins at *t*=2*bl* and finishes at time *t*_*R*_ which depends solely on the value of the ration *R*=*a*/*b*, and can in fact be shown to be the arrival time of an elastic wave propagating with the Rayleigh wave speed; i.e. *t*_*R*_=2*l*/*c*_*R*_ where *c*_*R*_ is the Rayleigh wave speed. This is because *F*_*x*_(*t*) and the *σ*_*xz*_ stress component of an edge dislocation vanish along the epicentral line only for the Rayleigh wave.

Once the Rayleigh wave arrives at the dislocation core, the image force becomes attractive again, and it is immediate to prove that *F*_*x*_ converges to its elastostatic value, because

5.8 $$\lim_{\kappa \to \infty }\left[\frac{1}{2(1-R^{2})}\left(\frac{R^{2}\kappa \sqrt{\kappa^{2}-4}}{2}H(\kappa -2)-\frac{{\left(R^{2}\kappa^{2}-2\right)}^{2}}{2R\kappa \sqrt{R^{2}\kappa^{2}-4}}H\left(\kappa -\frac{2}{R}\right)\right)\right]=\frac{1}{2}$$

and therefore the elastostatic solution (equation (1.1)) is recovered in that limit.

## 6. Image force on an injected, moving edge dislocation

As was described by Gurrutxaga-Lerma *et al.* [3], and in a manner similar to the solution for a non-uniformly moving screw dislocation, the elastodynamic fields of an injected, non-uniformly moving edge dislocation are formed by superposition of an injection contribution and an additional contribution describing the non-uniform motion. The injection contribution corresponds with the fields of an injected, quiescent edge dislocation, and was obtained by Gurrutxaga-Lerma *et al.* [3]. The mobile contributions were derived by Markenscoff & Clifton [5].

The problem of image forces for injected quiescent edge dislocations was solved in §5. The image force for the moving edge dislocations is modelled employing the image dislocation construction shown in figure 1*d*; the dislocation is modelled at a distance *l* from the free surface, and gliding towards the free surface along the *x*-axis with past history function *t*=*η*(*x*) (or *x*=*l*(*t*)); the image dislocation is a dislocation of same magnitude but opposite sign, located at a distance *l* from the surface in the initial instant, that glides towards the surface with the same past history function.

Under the image dislocation construction, it is trivial to check that all stress components cancel at the free surface except for, again, the *σ*_*xz*_ stress component. As in §5, the *σ*_*xz*_ stress distribution at the surface leads to the need of solving a Lamb's problem analogous to the injected, quiescent dislocation's. Following the formulation employed in §5, the free surface's contribution to the *σ*_*xz*_ component of stress will be equation (4.25)

$$\sigma_{xz}(x,z,t)=\int_{-\infty }^{\infty }\int_{0}^{\infty }[G(x,\varsigma -z,\vartheta -t)\zeta (\varsigma ,\vartheta )]\;\text{d}\vartheta \;\text{d}\varsigma ,$$

where all that changes in this case is the mathematical form of *ζ*(*z*,*t*), i.e. the distribution of *σ*_*xz*_ on the free surface is due to the superposition of the image dislocation and the actual dislocation.

For the case of a non-unifomly moving edge dislocation, the form of *ζ*(*z*,*t*) can be found from the *σ*_*xz*_ component of stress of a non-uniformly moving edge dislocation. It is given by (see [3]):

6.1 $$\begin{array}{rl} \zeta (z,t) & =\mu \frac{4B}{\pi b^{2}}\frac{\partial }{\partial t}\int_{0}^{\infty }H(\tilde{t}-\tilde{r}a)\frac{a^{4}{\tilde{x}}^{2}z^{2}{\tilde{r}}^{4}-{\tilde{T}}_{a}^{2}(8{\tilde{t}}^{2}{\tilde{x}}^{2}z^{2}-{\tilde{r}}^{4}{\tilde{t}}^{2})}{{\tilde{T}}_{a}{\tilde{r}}^{8}}\;\text{d}\xi \\ & \;-\mu \frac{B}{\pi b^{2}}\frac{\partial }{\partial t}\int_{0}^{\infty }H(\tilde{t}-\tilde{r}b)\frac{b^{4}{\left({\tilde{x}}^{4}-z^{4}\right)}^{2}+{\tilde{T}}_{b}^{2}(8{\tilde{t}}^{2}{\tilde{x}}^{2}z^{2}-{\tilde{r}}^{4}{\tilde{t}}^{2})}{{\tilde{T}}_{b}{\tilde{r}}^{8}}\;\text{d}\xi , \end{array}$$

where $\tilde{x}=l-\xi$, $\tilde{r}=\sqrt{{\tilde{x}}^{2}+z^{2}}$, ${\tilde{T}}_{a}=\sqrt{{\tilde{t}}^{2}-a^{2}{\tilde{r}}^{2}}$ and ${\tilde{T}}_{b}=\sqrt{{\tilde{t}}^{2}-b^{2}{\tilde{r}}^{2}}$. This form of the stress distribution at the free surface is slightly problematic because the order of integration and differentiation cannot be freely interchanged due to the presence of $T_{a}^{-3}$ non-integrable singularities. As described in [3,5], by rearranging terms and integrating by parts one can achieve the following working form:

6.2 $$\begin{array}{rl} \zeta (z,t) & =\mu \frac{4B}{\pi b^{2}}\{\int_{0}^{\infty }H(\tilde{t}-\tilde{r}a)\frac{-\tilde{t}({\tilde{x}}^{4}-6{\tilde{x}}^{2}z^{2}+z^{4})(3{\tilde{t}}^{2}-2a^{2}{\tilde{r}}^{2})}{{\tilde{r}}^{8}{\tilde{T}}_{a}}\;\text{d}\xi \\ & \;-\frac{a^{6}x^{2}z^{2}(-tx+r^{2}\eta^{\prime }(0))}{r^{4}T_{a}{\left(a^{2}x-t\eta^{\prime }(0)\right)}^{2}}H(t-ar)-\int_{0}^{\infty }H(\tilde{t}-a\tilde{r})\frac{\partial }{\partial t}\left[{\tilde{T}}_{a}\frac{\partial }{\partial \xi }\left[\frac{a^{4}z^{2}{\tilde{x}}^{2}}{{\tilde{r}}^{4}(a^{2}\tilde{x}-\tilde{t}\eta^{\prime }(\xi ))}\right]\right]\text{d}\xi \}\\ & \;-\mu \frac{B}{\pi b^{2}}\{\int_{0}^{\infty }H(\tilde{t}-\tilde{r}b)\frac{4\tilde{t}({\tilde{x}}^{4}-6{\tilde{x}}^{2}z^{2}+z^{4})(3{\tilde{t}}^{2}-2b^{2}{\tilde{r}}^{2})}{{\tilde{r}}^{8}{\tilde{T}}_{b}}\;\text{d}\xi \\ & \;-\frac{b^{4}{\left(x^{2}-z^{2}\right)}^{2}T_{b}}{r^{4}(b^{2}x-\eta^{\prime }(0)t)}H(t-br)+\int_{0}^{\infty }H(\tilde{t}-b\tilde{r})\frac{\partial }{\partial t}\left[{\tilde{T}}_{b}\frac{\partial }{\partial \xi }\left[\frac{b^{4}{\left({\tilde{x}}^{2}-z^{2}\right)}^{2}}{{\tilde{r}}^{4}(b^{2}\tilde{x}-\tilde{t}\eta^{\prime }(\xi ))}\right]\right]\text{d}\xi \}. \end{array}$$

Similar to the injected, quiescent dislocation's case, here *ζ*(*z*,*t*) is even with respect to *z*, so the convolution integral along the epicentral line must vanish.

Thus, for the non-uniformly moving edge dislocation, the image force will be given by

6.3 $$\begin{array}{rl} F_{x}(t) & =-\frac{\mu B^{2}}{2\pi b^{2}}\left[-\frac{t\sqrt{t^{2}-4a^{2}l^{2}}}{2l^{3}}H(t-2al)-\frac{{\left(t^{2}-2b^{2}l^{2}\right)}^{2}}{2\;l^{3}t\sqrt{t^{2}-4b^{2}l^{2}}}H(t-2bl)\right]\\ & \;+\mu \frac{4B^{2}}{\pi b^{2}}\frac{\partial }{\partial t}\int_{0}^{\infty }H(\tilde{t}-|\tilde{x}|a)\frac{{\tilde{T}}_{a}{\tilde{t}}^{2}}{{\tilde{x}}^{4}}\;\text{d}\xi -\mu \frac{B^{2}}{\pi b^{2}}\frac{\partial }{\partial t}\int_{0}^{\infty }H(\tilde{t}-|\tilde{x}|b)\frac{b^{4}{\tilde{x}}^{4}-{\tilde{T}}_{b}^{2}{\tilde{t}}^{2}}{{\tilde{T}}_{b}{\tilde{x}}^{4}}\;\text{d}\xi , \end{array}$$

where $\tilde{x}=2l-\xi$. Clearly, the image force is expressed in terms of two waves, the longitudinal and transverse waves, and is dependent on the past history of the dislocation (or its image's).

As seen for the screw dislocation, the case of the uniformly moving edge dislocation is more revealing. Consider the edge dislocation moving uniformly with speed *v*=1/*d*; the image force is found to be

6.4 $$F_{x}(t)=\frac{\mu B^{2}}{2\pi b^{2}}\left[d\frac{{\left(t^{2}-2b^{2}l^{2}\right)}^{2}}{l^{2}t\sqrt{t^{2}-4b^{2}l^{2}}(t-2dl)}H(t-2bl)-d\frac{t\sqrt{t^{2}-4a^{2}l^{2}}}{l^{2}(t-2dl)}H(t-2al)\right].$$

It might be more revealing to express it in non-dimensional terms as follows:

6.5 $$\begin{array}{r} F_{x}(\kappa )=\frac{\mu B^{2}}{2(1-\nu )\pi }\left[\frac{1}{l}\right]\underset{\mathrm{Dynamic}\;\mathrm{term}}{\underbrace{\left[\frac{1}{2(R^{2}-1)}\left(\frac{R^{2}\kappa \sqrt{\kappa^{2}-4}}{\left(M_{t}R\kappa -2\right)}H(\kappa -2)+\frac{{\left(R^{2}\kappa^{2}-2\right)}^{2}}{R\kappa \sqrt{R^{2}\kappa^{2}-4}(M_{t}R\kappa -2)}H\left(\kappa -\frac{2}{R}\right)\right)\right]}}, \end{array}$$

where *R*=*a*/*b* and *M*_*t*_=*b*/*d*=*v*/*c*_*t*_ is the transverse Mach number with respect to the dislocation's speed.

**Figure 5.**
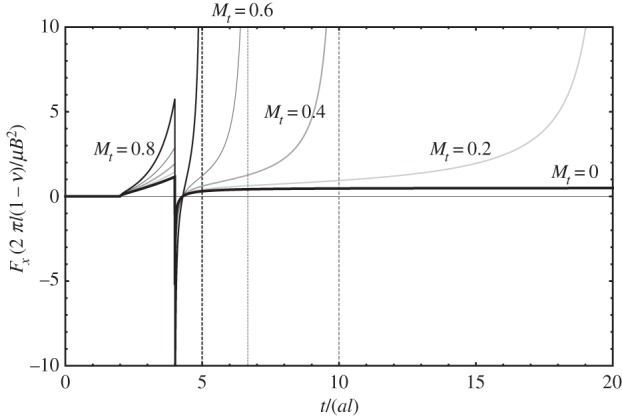
Magnitude of the image force for a uniformly moving edge dislocation. Here *R*=*a*/*b*=0.5. The dashed vertical lines signal the instant in time the dislocation reaches the free surface, for each dislocation speed.

The image force is represented in figure 5. As can be seen, for *M*_*t*_=0 the solution for the injected dislocation shown in figure 4 is recovered. As in that case, the 1/*l* term in the first bracket in equation (6.5) is the asymptotic value towards which the image force converges, but in this case only if *M*_*t*_=0. For *M*_*t*_>0, the situation is analogous to the case of the moving screw dislocation: the term in the second bracket in equation (6.5), representing the elastodynamic contribution to the image force, inevitably diverges. This is clearly seen, for various *M*_*t*_ values, in figure 5.

Unlike in the case of screw dislocations, edge dislocations are influenced by the longitudinal wavefront first, which in all cases prescribes a force of increasing magnitude with time until the arrival of the transverse wavefront at time *t*=4*al*. Thereafter, the dislocation experiences a brief transient reversal of the sign of the image force; this reversal is analogous to the case of the injected, quiescent edge dislocation. As can be seen in figure 5, the transient reversal occurs between *t*=2*bl* and *t*_*R*_, which can be shown to be independent of *d* and to correspond with the arrival time of the reflected Rayleigh wave (i.e. *t*_*R*_=2 *l*/*c*_*R*_). After the arrival of the Rayleigh wave, and unlike the quiescent dislocation's case, the image force quickly increases in magnitude with time, diverging when the dislocation reaches the free surface. The reversal of the sign of the image force in between the arrival of the transverse and the Rayleigh waves is significant, as it suggests that dislocations could reverse their way towards the surface, and move inwards towards the bulk instead.

**Figure 6.**
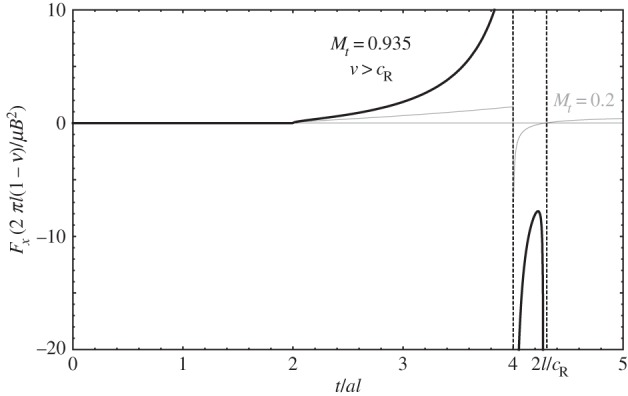
Image force for an injected dislocation moving uniformly with speed *v*=0.935*c*_*t*_, slightly larger than the Rayleigh wave speed, *c*_*R*_. Here *R*=*a*/*b*=0.5, so *c*_*R*_=0.9325*c*_*t*_.

The case of an edge dislocation moving uniformly with speed greater than the Rayleigh wave speed, *c*_*R*_, merits special consideration. As can be seen in figure 6, after the transverse wavefront reaches the dislocation at *t*=2*bl*, the image force reverses its sign, becoming repulsive. This is because the transverse component of *σ*_*xz*_ reverses its sign ahead of the dislocation's core for speeds larger than *c*_*R*_. This entails that after the arrival of the transverse wavefront, the dislocation can experience only a repulsive image force from the surface, which would suggest that such dislocation would not be able to reach the surface.

This seems unlikely. On one hand, a dislocation experiencing a repulsive image force would invariably tend to decelerate below the Rayleigh wave speed, at which point it could potentially reach the surface. Furthermore, as pointed out in [1], above the Rayleigh wave speed the dislocation's core may become unstable, leading to kinematic generation of dislocations rather than to dislocations moving faster than the Rayleigh wave speed.

Moreover, as postulated in [1], in the presence of a free surface, a dislocation moving faster than the Rayleigh wave speed would resonate with the surface, making the system's elastic energy diverge; this would prevent the dislocation from acquiring such speed. The solution procedure detailed in §4 agrees with this: as can be seen in equation (4.24), *Σ*_*xz*_ (i.e. the *σ*_*xz*_ stress field contribution due to the image fields in Laplace space), diverges when the Rayleigh function *R*(λ) vanishes. One of the roots of *R*(λ)=0 is the Rayleigh wave speed; hence, when *v*=*c*_*R*_, the *σ*_*xz*_ stress field of the moving dislocation in the presence of a free surface diverges, and so must the elastic energy of the system. In that way, the formulation presented here shows that in the presence of a free surface the Rayleigh wave speed is a limiting speed of edge dislocations because, beyond possible core instabilities, the elastic energy of the system diverges.

Finally, as in the screw dislocation's case, it is easy to check that the dynamic effects on the image force increase its magnitude significantly even at very low speeds and within the timescale and expected dislocation speeds of a quasi-static discrete dislocation dynamics simulation.

## 7. Conclusion

This article reports the derivation of the elastodynamic image forces experienced by straight edge and screw dislocations, both injected and either quiescent or moving. Closed-form, explicit formulae of the image force for these four cases have been obtained.

The image forces computed here display features characteristic of an elastodynamic description of the dislocation. For instance, the image forces are affected by marked retardation effects; the dislocations have been shown not to feel the presence of the free surface until their elastodynamic fields have had time to reach the free surface and be reflected back. This retardation time can be significant; for example, screw dislocations moving towards the free surface at the transverse speed of sound will not experience an image force at all.

Interestingly, the solutions presented here for moving dislocations diverge significantly both from the elastostatic prediction and those derived here for the case of injected, quiescent dislocations. Once a speed has been imparted on the dislocation, the image force is shown invariably to increase far beyond the asymptotic values obtained for non-moving dislocations. This increase in the magnitude of the elastodynamic image force is an inherently dynamic feature of our solutions, and is relevant even at relatively low dislocation speeds. For instance, for dislocations moving with a speed of the order of 100–200 m s^−1^, it is estimated that the image force will double its magnitude with respect to the elastostatic image force within 1–5 ns. These speeds and timescales are easily achievable in quasi-static applications of plasticity. The reported magnification is a consequence not of the injection process, but rather of the motion of the dislocation, and will remain even if the latter were pre-existing.

Thus, the results presented here suggests that dislocations will tend to accelerate towards the surface at a much faster rate than that predicted by elastostatics. Whether or not this dynamic effect could alter results of quasi-static dislocation dynamics, which only account for the elastostatic image force increasing in proportion to 1/*l*, is a topic worthy of further investigation. Furthermore, the image force of a moving edge dislocation has been shown to display anomalous behaviour for dislocations moving faster than the Rayleigh wave speed, as in that case the image force was shown to reverse its sign and become repulsive. In the presence of a free surface, the Rayleigh wave speed has usually been assumed to be the limiting speed of edge dislocations because the dislocation would resonate with the free surface; the results presented here are in agreement with this.

## Supplementary Material

SUPPLEMENTARY MATERIAL TO ‘ELASTODYNAMIC IMAGE FORCES OVER DISLOCATIONS’

## Footnotes

1 The displacement field, derived using the same procedure, is given by

2.18 $$\begin{array}{ll} u_{y}(x,z,t) & =\frac{B}{2\pi }[\tan^{-1}\left(\frac{\sqrt{t^{2}-b^{2}(x^{2}+z^{2})}}{t}\right)+\tan^{-1}\left(\frac{tz}{x\sqrt{t^{2}-b^{2}(x^{2}+z^{2})}}\right)\\ & +\frac{bx}{\sqrt{b^{2}x^{2}-2t^{2}}}\tanh^{-1}\left(\frac{btz}{\sqrt{b^{2}x^{2}-2t^{2}}\sqrt{t^{2}-b^{2}(x^{2}+z^{2})}}\right)]H(t-br). \end{array}$$

2 Note that the rotation of the coordinate system suffices to change the sign of the dislocation; i.e. the image dislocation is positive with respect to its local axes, but negative with respect to the actual dislocation's.

## Data accessibility

This work does not have any experimental data.
