# Supplementary material for: Elastodynamic image forces on dislocations
Source: Proc Math Phys Eng Sci. 2015 Sep 8;471(2181):20150433. doi: 10.1098/rspa.2015.0433 (PMC4614443; doi:10.1098/rspa.2015.0433)
Supplement: SUPPLEMENTARY MATERIAL TO ‘ELASTODYNAMIC IMAGE FORCES OVER DISLOCATIONS’ [file rspa20150433supp1.pdf]

SUPPLEMENTARY MATERIAL TO 'ELASTODYNAMIC IMAGE FORCES OVER DISLOCATIONS'

B. GURRUTXAGA-LERMA, D.S. BALINT, D.DINI, A.P. SUTTON

Explicit form of $G(x,z,t)$:

(1)
$$G(x,z,t)=\frac{1}{\pi}\left[H(t-ra)\frac{\mathrm{d}F_a(t)}{\mathrm{d}t}+H(t-rb)\frac{\mathrm{d}F_b(t)}{\mathrm{d}t}\right]$$

Define:

$$\frac{\mathrm{d}F_a(t)}{\mathrm{d}t}=\frac{D_{a1}}{N_{a1}}+\frac{D_{a2}}{N_{a2}}$$

$$\frac{\mathrm{d}F_b(t)}{\mathrm{d}t}=\frac{D_{b1}}{N_{b1}}+\frac{D_{b2}}{N_{b2}}+\frac{D_{b3}}{N_{b3}}$$

to find:

(2)
$$\begin{aligned}D_{a1}=\frac{1}{r^4}\Big\{&b^8r^{20}+8b^6r^{16}(tz+T_ax)(T_ax-tz)+8b^4r^{12}\left(3t^4z^4-2t^2T_a^2x^2z^2+3T_a^4x^4\right)+32b^2r^8\Upsilon_a^2(tz+T_ax)(T_ax-tz)\\&+8R_ar^2\left(T_az\sin(\theta_a)\left(b^4r^8\left(x^2\left(2t^2+T_a^2\right)-t^2z^2\right)+4b^2r^4\Upsilon_a^2-4\Upsilon_a^2\left(t^2\left(2x^2+z^2\right)-T_a^2x^2\right)\right)\right.\\&\left.+tx\cos(\theta_a)\left(b^4r^8\left(z^2\left(t^2+2T_a^2\right)-T_a^2x^2\right)-4b^2r^4\Upsilon_a^2-4\Upsilon_a^2\left(T_a^2\left(x^2+2z^2\right)-t^2z^2\right)\right)\right)\\&+16\Upsilon_a^2\left(R_a^2\left(t^2x^2+T_a^2z^2\right)+r^4\left(t^2z^2+T_a^2x^2\right)^2\right)\Big\}\end{aligned}$$

(3)
$$\begin{aligned}D_{a2}=r^{16}\Big\{&b^8+\frac{1}{r^4}\left[8b^6(tz+T_ax)(T_ax-tz)\right]\\&+\frac{1}{r^{10}}\left[8b^4\left(\varrho_aT_az\sin(\theta_a)\left(t^2\left(2x^2-z^2\right)+T_a^2x^2\right)+\varrho_atx\cos(\theta_a)\left(t^2z^2-T_a^2\left(x^2-2z^2\right)\right)\right.\right.\\&\left.\left.+r^2\left(3t^4z^4-2t^2T_a^2x^2z^2+3T_a^4x^4\right)\right)\right]+\frac{1}{r^{14}}\left[32b^2\Upsilon_a^2\left(-\varrho_atx\cos(\theta_a)+\varrho_aT_az\sin(\theta_a)+r^2\left(T_a^2x^2-t^2z^2\right)\right)\right]\\&+\frac{1}{r^{20}}\left[16\Upsilon_a^2\left(\varrho_a^2t^2x^2+\varrho_a^2T_a^2z^2+2\varrho_ar^2T_az\sin(\theta_a)\left(T_a^2x^2-t^2\left(2x^2+z^2\right)\right)\right.\right.\\&\left.\left.+2\varrho_ar^2tx\cos(\theta_a)\left(t^2z^2-T_a^2\left(x^2+2z^2\right)\right)+r^4t^4z^4+2r^4t^2T_a^2x^2z^2+r^4T_a^4x^4\right)\right]\Big\}^2\end{aligned}$$

(4)
$$\begin{aligned}N_{a1}=8\Bigg(&-8tT_a\Upsilon_a^2z\cos(\theta_a)\left(-a^2x^2+b^2\left(z^2-x^2\right)+t^2\right)\\&+x\left(r^4T_az\left(b^4r^4\left(x^2-z^2\right)\left(2t^2-a^2r^2\right)-4a^2\Upsilon_a^2+4b^2\Upsilon_a^2\right)+2\varrho_a\Upsilon_a^2\sin(\theta_a)\right)\\&+\frac{1}{r^2T_a}\Bigg[tx\Bigg(t\left(4T_a\Upsilon_a\sin(\theta_a)\left(2\Upsilon_ar^2\left(-a^2x^2+b^2\left(z^2-x^2\right)+t^2\right)+\varrho_ar^2(\Upsilon_a+2r^2)\right)\right.\\&\left.+r^6z\left(2b^2\left(b^2r^4\left(t^2+2T_a^2\right)\left(x^2-z^2\right)+8r^2T_a^2\Upsilon_a+2\Upsilon_a^2\right)-a^2\left(b^4r^6\left(x^2-z^2\right)+16r^2T_a^2\Upsilon_a+4\Upsilon_a^2\right)\right)\right)\\&+\frac{2\varrho_a\Upsilon_a^2r^2xz\cos(\theta_a)\left(\varpi_a\left(t^2+T_a^2\right)+2t^2T_a^2\left(z^2-x^2\right)\right)}{\varpi_a^2+4t^2T_a^2x^2z^2}\Bigg)\Bigg]\\&+\frac{2\varrho_a\Upsilon_a^2r^2xz^2\sin(\theta_a)\left(\varpi_a\left(t^2+T_a^2\right)+2t^2T_a^2\left(z^2-x^2\right)\right)}{r^2\left(\varpi_a^2+4t^2T_a^2x^2z^2\right)}-\frac{2\varrho_at\Upsilon_a^2r^2z\cos(\theta_a)}{r^2T_a}-4\varrho_atT_a\Upsilon_a^2z\cos(\theta_a)\\&-8\varrho_atT_a\Upsilon_ar^2z\cos(\theta_a)\Bigg)\end{aligned}$$

(5)

$$
\begin{aligned}
N_{a2} = -\frac{1}{r^{16}} \Bigg[64 \Bigg(tx \Bigg(& T_a z \left(b^4 \left(2t^2 - a^2 r^2\right)\left(x^2 - z^2\right) r^4 - 4a^2\Upsilon_a^2 + 4b^2\Upsilon_a^2\right) r^4 + \frac{2\varrho_a \Upsilon_a^2 \Xi_a \sin(\theta_a)}{r^2} \Bigg) \\
& - \frac{2\varrho_a T_a \Upsilon_a^2 \Xi_a z\cos(\theta_a)}{r^2}\Bigg) \Bigg(b^6 \left(\frac{tx}{T_a} - z\right)(T_a x + tz) r^{12} + b^6\left(\frac{tx}{T_a}+z\right)(T_a x - tz) r^{12} \\
& + b^4 \Bigg(12tT_a^2x^4 - 4t^3z^2x^2 - 4tT_a^2z^2x^2 \\
& + \frac{\varrho_a tz\left(2t^2T_a^2\left(x^2-z^2\right) - \theta_a\left(t^2+T_a^2\right)\right)\left(t^2z^2 - T_a^2\left(x^2-2z^2\right)\right)\sin(\theta_a)x^2}{r^2T_a\left(\theta_a^2 + 4t^2T_a^2x^2z^2\right)} \\
& + \frac{\varrho_a T_a z^2\left(\left(2x^2-z^2\right)t^2 + T_a^2x^2\right)\left(2t^2\left(z^2-x^2\right)T_a^2 + \theta_a\left(t^2+T_a^2\right)\right)\cos(\theta_a)x}{r^2T_a\left(\theta_a^2+4t^2T_a^2x^2z^2\right)} \\
& + \frac{4t^2\left(\left(z^2-x^2\right)b^2 + t^2 - a^2x^2\right)\left(t^2z^2 - T_a^2\left(x^2-2z^2\right)\right)\cos(\theta_a)x}{r^2} \\
& + \frac{\varrho_a\left(t^2z^2 - T_a^2\left(x^2-2z^2\right)\right)\cos(\theta_a)x}{r^2} - \frac{2\varrho_a t^2\left(x^2-3z^2\right)\cos(\theta_a)x}{r^2} + 12t^3z^4 \\
& + \frac{\varrho_a tz\left(\left(2x^2-z^2\right)t^2 + T_a^2x^2\right)\sin(\theta_a)}{r^2T_a} + \frac{4tT_a z\left(\left(2x^2-z^2\right)t^2+T_a^2x^2\right)\left(\left(z^2-x^2\right)b^2+t^2-a^2x^2\right)\sin(\theta_a)}{r^2} \\
& - \frac{2\varrho_a tT_a z\left(z^2-3x^2\right)\sin(\theta_a)}{r^2}\Bigg) r^8 + 4b^2\Bigg(-\frac{4x\left(\left(z^2-x^2\right)b^2+t^2-a^2x^2\right)\cos(\theta_a)t^2}{r^2} + 2x^2t - 2z^2t + \frac{\varrho_a z\sin(\theta_a)t}{r^2T_a} \\
& + \frac{4T_a z\left(\left(z^2-x^2\right)b^2+t^2-a^2x^2\right)\sin(\theta_a)t}{r^2} - \frac{\varrho_a x^2z\left(2t^2T_a^2\left(x^2-z^2\right) - \theta_a\left(t^2+T_a^2\right)\right)\sin(\theta_a)t}{r^2T_a\left(\theta_a^2+4t^2T_a^2x^2z^2\right)} \\
& + \frac{\varrho_a T_a xz^2\left(2t^2\left(z^2-x^2\right)T_a^2 + \theta_a\left(t^2+T_a^2\right)\right)\cos(\theta_a)}{r^2T_a\left(\theta_a^2+4t^2T_a^2x^2z^2\right)} - \frac{\varrho_a x\cos(\theta_a)}{r^2}\Bigg)\Upsilon_a^2r^4 \\
& - 16b^2t\Upsilon_a r^2\left(\left(t^2z^2 - T_a^2x^2\right)r^2 + \varrho_a tx\cos(\theta_a) - \varrho_a T_a z\sin(\theta_a)\right)r^2 \\
& + 4\Upsilon_a^2\Bigg(2tT_a^2x^4 + 2t^3z^2x^2 + 2tT_a^2z^2x^2 + \frac{\varrho_a^2tx^2}{r^4} + \frac{4\varrho_a t^3\left(\left(z^2-x^2\right)b^2+t^2-a^2x^2\right)x^2}{r^4} \\
& + \frac{\varrho_a tz\left(2t^2T_a^2\left(x^2-z^2\right) - \theta_a\left(t^2+T_a^2\right)\right)\left(t^2z^2 - T_a^2\left(x^2+2z^2\right)\right)\sin(\theta_a)x^2}{r^2T_a\left(\theta_a^2+4t^2T_a^2x^2z^2\right)} \\
& + \frac{\varrho_a T_a z^2\left(2t^2\left(z^2-x^2\right)T_a^2 + \theta_a\left(t^2+T_a^2\right)\right)\left(T_a^2x^2 - t^2\left(2x^2+z^2\right)\right)\cos(\theta_a)x}{r^2T_a\left(\theta_a^2+4t^2T_a^2x^2z^2\right)} \\
& + \frac{4t^2\left(\left(z^2-x^2\right)b^2+t^2-a^2x^2\right)\left(t^2z^2 - T_a^2\left(x^2+2z^2\right)\right)\cos(\theta_a)x}{r^2} \\
& + \frac{\varrho_a\left(t^2z^2 - T_a^2\left(x^2+2z^2\right)\right)\cos(\theta_a)x}{r^2} \\
& - \frac{2\varrho_a t^2r^2\cos(\theta_a)x}{r^2} + 2t^3z^4 + \frac{\varrho_a^2tz^2}{r^4} + \frac{4\varrho_a tT_a^2z^2\left(\left(z^2-x^2\right)b^2+t^2-a^2x^2\right)}{r^4} \\
& + \frac{\varrho_a tz\left(T_a^2x^2 - t^2\left(2x^2+z^2\right)\right)\sin(\theta_a)}{r^2T_a} \\
& - \frac{2\varrho_a tT_a zr^2\sin(\theta_a)}{r^2} - \frac{4tT_a z\left(\left(z^2-x^2\right)b^2+t^2-a^2x^2\right)\left(t^2\left(2x^2+z^2\right) - T_a^2x^2\right)\sin(\theta_a)}{r^2}\Bigg) \\
& + \frac{1}{r^4}\left[8t\Upsilon_a r^2\left(T_a^4x^4r^4 + t^4z^4r^4 + 2t^2T_a^2x^2z^2r^4 + 2\varrho_a tx\left(t^2z^2 - T_a^2\left(x^2+2z^2\right)\right)\cos(\theta_a)r^2\right.\right. \\
& \left.\left. + 2\varrho_a T_a z\left(T_a^2x^2 - t^2\left(2x^2+z^2\right)\right)\sin(\theta_a)r^2 + \varrho_a^2t^2x^2 + \varrho_a^2T_a^2z^2\right)\right]\Bigg)\Bigg]
\end{aligned}
$$

$$
\begin{aligned}
N_{b1} = -t \Bigg(tx \Bigg(& -b^8 r^{16} - 16 b^6 r^{12} T_b^2 z^2 - \frac{8 \varrho_b T_b \Xi_b z \sin(\theta_b) \left(b^4 r^8 - 4 \Upsilon_b^2 \right)}{r^2} + 8 b^4 r^8 \left(t^4 z^4 + 10 t^2 T_b^2 x^2 z^2 + T_b^4 x^4 \right) \\
& + 64 b^2 r^4 \left(t^2 T_b z^3 + \left(T_b^2 \right)^{3/2} x^2 z \right)^2 - 16 \Upsilon_b^4 \Bigg) \\
& - \frac{4 \varrho_b \Xi_b \cos(\theta_b) \left(b^4 r^8 \left(t^2 z^2 + T_b x^2 \right) + 4 b^2 r^4 \Upsilon_b^2 + 4 \Upsilon_b^2 \left(t^2 z^2 - T_b^2 x^2 \right) \right)}{r^2} \Bigg)
\end{aligned} \tag{6}
$$

$$
\begin{aligned}
& N_{b3} \\
& = -8 \Bigg(2 b^6 t x^2 r^{12} - 2 b^6 t z^2 r^{12} + 12 b^4 t T_b^2 x^4 r^8 + 12 b^4 t^3 z^4 r^8 - 4 b^4 t^3 x^2 z^2 r^8 - 4 b^4 t T_b^2 x^2 z^2 r^8 + 24 b^2 t T_b^4 x^6 r^4 - 24 b^2 t^5 z^6 r^4 \\
& - 8 b^2 t^5 x^2 z^4 r^4 - 16 b^2 t^3 T_b^2 x^2 z^4 r^4 + 8 b^2 t T_b^4 x^4 z^2 r^4 + 16 b^2 t^3 T_b^2 x^4 z^2 r^4 + 16 t T_b^6 x^8 + 16 t^7 z^8 + \frac{4 \varrho_b^2 t T_b^4 x^6}{r^4} + \frac{8 \varrho_b^2 t^3 T_b^2 x^6}{r^4} \\
& + \frac{4 \varrho_b^2 t^5 z^6}{r^4} + \frac{8 \varrho_b^2 t^3 T_b^2 z^6}{r^4} + 16 t^7 x^2 z^6 + 48 t^5 T_b^2 x^2 z^6 + 48 t^3 T_b^4 x^4 z^4 + 48 t^5 T_b^2 x^4 z^4 + \frac{12 \varrho_b^2 t^5 x^2 z^4}{r^4} + \frac{8 \varrho_b^2 t T_b^4 x^2 z^4}{r^4} \\
& + \frac{16 \varrho_b^2 t^3 T_b^2 x^2 z^4}{r^4} + 16 t T_b^6 x^6 z^2 + 48 t^3 T_b^4 x^6 z^2 + \frac{8 \varrho_b^2 t^5 x^4 z^2}{r^4} + \frac{12 \varrho_b^2 t T_b^4 x^4 z^2}{r^4} + \frac{16 \varrho_b^2 t^3 T_b^2 x^4 z^2}{r^4} \\
& + \frac{16 \varrho_b t^3 T_b^4 x^6 \left(\left(z^2 - x^2 \right) a^2 + t^2 - b^2 x^2 \right)}{r^4} + \frac{16 \varrho_b t^5 T_b^2 z^6 \left(\left(z^2 - x^2 \right) a^2 + t^2 - b^2 x^2 \right)}{r^4} \\
& + \frac{16 \varrho_b t^7 x^2 z^4 \left(\left(z^2 - x^2 \right) a^2 + t^2 - b^2 x^2 \right)}{r^4} + \frac{32 \varrho_b t^3 T_b^4 x^2 z^4 \left(\left(z^2 - x^2 \right) a^2 + t^2 - b^2 x^2 \right)}{r^4} \\
& + \frac{16 \varrho_b t T_b^6 x^4 z^2 \left(\left(z^2 - x^2 \right) a^2 + t^2 - b^2 x^2 \right)}{r^4} + \frac{32 \varrho_b t^5 T_b^2 x^4 z^2 \left(\left(z^2 - x^2 \right) a^2 + t^2 - b^2 x^2 \right)}{r^4} \\
& + \frac{1}{r^2 T_b \left(\varpi_b^2 + 4 t^2 T_b^2 x^2 z^2 \right)} \left[\varrho_b T_b x z^2 \left(\left(\varpi_b + 2 T_b T_b \left(z^2 - x^2 \right) \right) t^2 + T_b T_b \varpi_b \right) \left(b^4 \left(\left(2 x^2 - z^2 \right) t^2 + T_b^2 x^2 \right) r^8 \right. \right. \\
& \left. \left. + 4 b^2 \Upsilon_b^2 r^4 - 4 \Upsilon_b^2 \left(\left(2 x^2 + z^2 \right) t^2 + T_b x^2 \right) \right) \cos(\theta_b) \right] \\
& - \frac{\varrho_b x \left(b^4 \left(T_b^2 \left(x^2 - 2 z^2 \right) - t^2 z^2 \right) r^8 + 4 b^2 \Upsilon_b^2 r^4 + 4 \Upsilon_b^2 \left(T_b^2 \left(x^2 + 2 z^2 \right) - t^2 z^2 \right) \right) \cos(\theta_b)}{r^2} \\
& + \frac{4 t^2 x \left(\left(z^2 - x^2 \right) a^2 + t^2 - b^2 x^2 \right) \left(b^4 \left(t^2 z^2 - T_b^2 \left(x^2 - 2 z^2 \right) \right) r^8 - 4 b^2 \Upsilon_b^2 r^4 - 4 \Upsilon_b^2 \left(T_b^2 \left(x^2 + 2 z^2 \right) - t^2 z^2 \right) \right) \cos(\theta_b)}{r^2} \\
& - \frac{2 \varrho_b t^2 x \left(b^4 \left(x^2 - 3 z^2 \right) r^8 + 8 b^2 \Upsilon_b r^6 + 4 \Upsilon_b r^2 \left(2 \left(x^2 + 2 z^2 \right) T_b^2 - 2 t^2 z^2 + \Upsilon_b \right) \right) \cos(\theta_b)}{r^2} \\
& - \frac{4 t T_b z \left(\left(z^2 - x^2 \right) a^2 + t^2 - b^2 x^2 \right) \left(b^4 \left(t^2 \left(z^2 - 2 x^2 \right) - T_b^2 x^2 \right) r^8 - 4 b^2 \Upsilon_b^2 r^4 + 4 \Upsilon_b^2 \left(\left(2 x^2 + z^2 \right) t^2 + T_b x^2 \right) \right) \sin(\theta_b)}{r^2} \\
& + \frac{\varrho_b t z \left(b^4 \left(\left(2 x^2 - z^2 \right) t^2 + T_b^2 x^2 \right) r^8 + 4 b^2 \Upsilon_b^2 r^4 - 4 \Upsilon_b^2 \left(\left(2 x^2 + z^2 \right) t^2 + T_b x^2 \right) \right) \sin(\theta_b)}{r^2 T_b} \\
& + \frac{1}{r^2 T_b \left(\varpi_b^2 + 4 t^2 T_b^2 x^2 z^2 \right)} \left[\varrho_b t x^2 z \left(- \left(\varpi_b + 2 T_b T_b \left(z^2 - x^2 \right) \right) t^2 - T_b T_b \varpi_b \right) \left(b^4 \left(t^2 z^2 - T_b^2 \left(x^2 - 2 z^2 \right) \right) r^8 - 4 b^2 \Upsilon_b^2 r^4 \right. \right. \\
& \left. \left. - 4 \Upsilon_b^2 \left(T_b^2 \left(x^2 + 2 z^2 \right) - t^2 z^2 \right) \right) \sin(\theta_b) \right] \\
& - \frac{2 \varrho_b t T_b z \left(b^4 \left(z^2 - 3 x^2 \right) r^8 - 8 b^2 \Upsilon_b r^6 + 4 \Upsilon_b r^2 \left(\Upsilon_b + 2 \left(\left(2 x^2 + z^2 \right) t^2 + T_b x^2 \right) \right) \right) \sin(\theta_b)}{r^2} \Bigg) \cdot \\
& \cdot \Bigg(t x \Bigg(- b^8 r^{16} - 16 b^6 T_b^2 z^2 r^{12} + 8 b^4 \left(T_b^4 x^4 + 10 t^2 T_b^2 z^2 x^2 + t^4 z^4 \right) r^8 \\
& + 64 b^2 \left(x^2 z \left(T_b^2 \right)^{3/2} + t^2 T_b z^3 \right)^2 r^4 - 16 \Upsilon_b^4 - \frac{8 \varrho_b T_b \left(b^4 r^8 - 4 \Upsilon_b^2 \right) \Xi_b z \sin(\theta_b)}{r^2} \Bigg) \\
& - \frac{4 \varrho_b \Xi_b \left(b^4 \left(T_b x^2 + t^2 z^2 \right) r^8 + 4 b^2 \Upsilon_b^2 r^4 + 4 \Upsilon_b^2 \left(t^2 z^2 - T_b^2 x^2 \right) \right) \cos(\theta_b)}{r^2} \Bigg)
\end{aligned} \tag{7}
$$

$$
\begin{aligned}
N_{b3} = r^2 T_b \Bigg(& b^8 r^{16} - 8b^6 r^{12} t^2 z^2 + 8b^6 r^{12} T_b^2 x^2 + 24b^4 r^8 t^4 z^4 - 16b^4 r^8 t^2 T_b^2 x^2 z^2 + 24b^4 r^8 T_b^4 x^4 - 32b^2 r^4 t^6 z^6 \\
& - 32b^2 r^4 t^4 T_b^2 x^2 z^4 + 32b^2 r^4 t^2 T_b^4 x^4 z^2 + 32b^2 r^4 T_b^6 x^6 \\
& + \frac{8\varrho_b T_b z \sin(\theta_b) \left(b^4 r^8 \left(t^2 \left(2x^2 - z^2\right) + T_b^2 x^2\right) + 4b^2 r^4 \Upsilon_b^2 - 4\Upsilon_b^2 \left(t^2 \left(2x^2 + z^2\right) + T_b x^2\right)\right)}{r^2} \\
& + \frac{8\varrho_b t x \cos(\theta_b) \left(b^4 r^8 \left(t^2 z^2 - T_b^2 \left(x^2 - 2z^2\right)\right) - 4b^2 r^4 \Upsilon_b^2 - 4\Upsilon_b^2 \left(T_b^2 \left(x^2 + 2z^2\right) - t^2 z^2\right)\right)}{r^2} + \frac{16\varrho_b^2 t^6 x^2 z^4}{r^4} \\
& + \frac{32\varrho_b^2 t^4 T_b^2 x^4 z^2}{r^4} + \frac{16\varrho_b^2 t^4 T_b^2 z^6}{r^4} + \frac{16\varrho_b^2 t^2 T_b^4 x^6}{r^4} + \frac{32\varrho_b^2 t^2 T_b^4 x^2 z^4}{r^4} + \frac{16\varrho_b^2 T_b^6 x^4 z^2}{r^4} + 16t^8 z^8 + 64t^6 T_b^2 x^2 z^6 \\
& + 96t^4 T_b^4 x^4 z^4 + 64t^2 T_b^6 x^6 z^2 + 16T_b^8 x^8 \Bigg)
\end{aligned} \tag{8}
$$

$$
\begin{aligned}
D_{b1} = \frac{T_b^3}{r^2} \big\{ & b^8 r^{20} - 8b^6 r^{16} t^2 z^2 + 8b^6 r^{16} T_b^2 x^2 + 24b^4 r^{12} t^4 z^4 - 16b^4 r^{12} t^2 T_b^2 x^2 z^2 + 24b^4 r^{12} T_b^4 x^4 - 32b^2 r^8 t^6 z^6 \\
& - 32b^2 r^8 t^4 T_b^2 x^2 z^4 + 32b^2 r^8 t^2 T_b^4 x^4 z^2 + 32b^2 r^8 T_b^6 x^6 \\
& + 8\varrho_b r^2 T_b z \sin(\theta_b) \left(b^4 r^8 \left(t^2 \left(2x^2 - z^2\right) + T_b^2 x^2\right) + 4b^2 r^4 \Upsilon_b^2 - 4\Upsilon_b^2 \left(t^2 \left(2x^2 + z^2\right) + T_b x^2\right)\right) \\
& + 8\varrho_b r^2 t x \cos(\theta_b) \left(b^4 r^8 \left(t^2 z^2 - T_b^2 \left(x^2 - 2z^2\right)\right) - 4b^2 r^4 \Upsilon_b^2 - 4\Upsilon_b^2 \left(T_b^2 \left(x^2 + 2z^2\right) - t^2 z^2\right)\right) + 16\varrho_b^2 t^6 x^2 z^4 \\
& + 32\varrho_b^2 t^4 T_b^2 x^4 z^2 + 16\varrho_b^2 t^4 T_b^2 z^6 + 16\varrho_b^2 t^2 T_b^4 x^6 + 32\varrho_b^2 t^2 T_b^4 x^2 z^4 + 16\varrho_b^2 T_b^6 x^4 z^2 + 16r^4 t^8 z^8 + 64r^4 t^6 T_b^2 x^2 z^6 \\
& + 96r^4 t^4 T_b^4 x^4 z^4 + 64r^4 t^2 T_b^6 x^6 z^2 + 16r^4 T_b^8 x^8 \big\}
\end{aligned} \tag{9}
$$

$$
\begin{aligned}
D_{b2} = \frac{T_b^2}{r^6} \big(& b^8 r^{20} - 8b^6 r^{16} t^2 z^2 + 8b^6 r^{16} T_b^2 x^2 + 24b^4 r^{12} t^4 z^4 - 16b^4 r^{12} t^2 T_b^2 x^2 z^2 + 24b^4 r^{12} T_b^4 x^4 - 32b^2 r^8 t^6 z^6 \\
& - 32b^2 r^8 t^4 T_b^2 x^2 z^4 + 32b^2 r^8 t^2 T_b^4 x^4 z^2 + 32b^2 r^8 T_b^6 x^6 \\
& + 8\varrho_b r^2 T_b z \sin(\theta_b) \left(b^4 r^8 \left(t^2 \left(2x^2 - z^2\right) + T_b^2 x^2\right) + 4b^2 r^4 \Upsilon_b^2 - 4\Upsilon_b^2 \left(t^2 \left(2x^2 + z^2\right) + T_b x^2\right)\right) \\
& + 8\varrho_b r^2 t x \cos(\theta_b) \left(b^4 r^8 \left(t^2 z^2 - T_b^2 \left(x^2 - 2z^2\right)\right) - 4b^2 r^4 \Upsilon_b^2 - 4\Upsilon_b^2 \left(T_b^2 \left(x^2 + 2z^2\right) - t^2 z^2\right)\right) + 16\varrho_b^2 t^6 x^2 z^4 \\
& + 32\varrho_b^2 t^4 T_b^2 x^4 z^2 + 16\varrho_b^2 t^4 T_b^2 z^6 + 16\varrho_b^2 t^2 T_b^4 x^6 + 32\varrho_b^2 t^2 T_b^4 x^2 z^4 + 16\varrho_b^2 T_b^6 x^4 z^2 + 16r^4 t^8 z^8 + 64r^4 t^6 T_b^2 x^2 z^6 \\
& + 96r^4 t^4 T_b^4 x^4 z^4 + 64r^4 t^2 T_b^6 x^6 z^2 + 16r^4 T_b^8 x^8 \big)^2
\end{aligned} \tag{10}
$$

(11)

$$D_{b3} = \Bigg(-b^8r^{16} - 16b^6T_b^2z^2r^{12} + 8b^4\left(T_b^4x^4 + 10t^2T_b^2z^2x^2 + t^4z^4\right)r^8 + 64b^2\left(x^2z\left(T_b^2\right)^{3/2} + t^2T_bz^3\right)^2r^4 - 16\Upsilon_b^4 - \frac{8\varrho_bT_b\left(b^4r^8 - 4\Upsilon_b^2\right)\Xi_bz\sin(\theta_b)}{r^2}\Bigg)x$$
$$+ 8\Bigg(-4b^6tz^2r^{12} + 4b^4t\left(\left(x^4 + 5z^2x^2\right)T_b^2 + t^2z^2\left(5x^2 + z^2\right)\right)r^8 + 16b^2\left(x^2z\left(T_b^2\right)^{3/2} + t^2T_bz^3\right)\left(\frac{t^3z^3}{T_b} + tT_b\left(3x^2 + 2z^2\right)z\right)r^4 - 16t\Upsilon_b^3\left(x^2 + z^2\right)$$
$$+ \frac{16\varrho_btT_b\Upsilon_b\Xi_bzr^2\sin(\theta_b)}{r^2} + \frac{4tT_b\left(b^4r^8 - 4\Upsilon_b^2\right)\Xi_bz\left(\left(x^2 - z^2\right)a^2 - t^2 + b^2x^2\right)\sin(\theta_b)}{r^2}$$
$$- \frac{2\varrho_btT_b\left(b^4r^8 - 4\Upsilon_b^2\right)zr^2\sin(\theta_b)}{r^2} - \frac{\varrho_bt\left(b^4r^8 - 4\Upsilon_b^2\right)\Xi_bz\sin(\theta_b)}{r^2T_b}$$
$$- \frac{\varrho_bT_b\left(b^4r^8 - 4\Upsilon_b^2\right)x\Xi_bz^2\left(\left(\varpi_b + 2T_bT_b\left(z^2 - x^2\right)\right)t^2 + T_bT_b\varpi_b\right)\cos(\theta_b)}{r^2T_b\left(\varpi_b^2 + 4t^2T_b^2x^2z^2\right)}\Bigg)tx$$
$$- \frac{8\varrho_btr^2\left(b^4\left(T_bx^2 + t^2z^2\right)r^8 + 4b^2\Upsilon_b^2r^4 + 4\Upsilon_b^2\left(t^2z^2 - T_b^2x^2\right)\right)\cos(\theta_b)}{r^2}$$
$$- \frac{16t\Xi_b\left(\left(z^2 - x^2\right)a^2 + t^2 - b^2x^2\right)\left(b^4\left(T_bx^2 + t^2z^2\right)r^8 + 4b^2\Upsilon_b^2r^4 + 4\Upsilon_b^2\left(t^2z^2 - T_b^2x^2\right)\right)\cos(\theta_b)}{r^2}$$
$$- \frac{8\varrho_bt\Xi_b\left(b^4\left(z^2 - x^2\right)r^8 + 8b^2\Upsilon_br^6 + 4\Upsilon_b\left(\Upsilon_b\left(z^2 - x^2\right) - 2r^2\left(T_b^2x^2 - t^2z^2\right)\right)\right)\cos(\theta_b)}{r^2}$$
$$- \frac{4\varrho_bx\Xi_bz\left(b^4\left(T_bx^2 + t^2z^2\right)r^8 + 4b^2\Upsilon_b^2r^4 + 4\Upsilon_b^2\left(t^2z^2 - T_b^2x^2\right)\right)\left(-\left(\varpi_b + 2T_bT_b\left(z^2 - x^2\right)\right)t^2 - T_bT_b\varpi_b\right)\sin(\theta_b)}{r^2T_b\left(\varpi_b^2 + 4t^2T_b^2x^2z^2\right)}$$

Where:

$$r^2 = x^2 + z^2$$
$$T_a = \sqrt{t^2 - a^2r^2},$$
$$T_b = \sqrt{t^2 - b^2r^2},$$
$$\Upsilon_a = Ta^2x^2 + t^2z^2$$
$$\Upsilon_b = Tb^2x^2 + t^2z^2$$
$$\Xi_a = t^2x^2 + T_a^2z^2$$
$$\Xi_b = t^2x^2 + T_b^2z^2$$
$$\varrho_a = t^2\left(x^2 - z^2\right) - r^2\left(a^2x^2 - b^2r^2\right)$$
$$\varrho_b = -2t^2\left(a^2\left(x^2 - z^2\right) + b^2x^2\right) - a^2r^2 + b^2x^2 + t^4$$
$$\varpi_a = (-r^2(-b^2r^2 + a^2x^2) + t^2(x^2 - z^2))$$
$$\varpi_a = (-r^2(-a^2r^2 + b^2x^2) + t^2(x^2 - z^2))$$
$$\tan\left[2\theta_a\right] = \frac{2txz\sqrt{t^2 - a^2r^2}}{t^2\left(x^2 - z^2\right) - r^2\left(a^2x^2 - b^2r^2\right)}$$
$$\tan\left[2\theta_b\right] = \frac{2txz\sqrt{t^2 - b^2r^2}}{t^2\left(x^2 - z^2\right) - r^2\left(b^2x^2 - a^2r^2\right)}$$
